# NMR as a “Gold Standard” Method in Drug Design and Discovery

**DOI:** 10.3390/molecules25204597

**Published:** 2020-10-09

**Authors:** Abdul-Hamid Emwas, Kacper Szczepski, Benjamin Gabriel Poulson, Kousik Chandra, Ryan T. McKay, Manel Dhahri, Fatimah Alahmari, Lukasz Jaremko, Joanna Izabela Lachowicz, Mariusz Jaremko

**Affiliations:** 1 Core Labs, King Abdullah University of Science and Technology, Thuwal 23955-6900, Saudi Arabia; 2 Biological and Environmental Sciences & Engineering Division (BESE), King Abdullah University of Science and Technology (KAUST), Thuwal 23955-6900, Saudi Arabia; kacper.szczepski@kaust.edu.sa (K.S.); benjamingabriel.poulson@kaust.edu.sa (B.G.P.); kousik.chandra@kaust.edu.sa (K.C.); lukasz.jaremko@kaust.edu.sa (L.J.); 3 Department of Chemistry, University of Alberta, Edmonton, AB T6G 2W2, Canada; ryan.mckay@ualberta.ca; 4 Biology Department, Faculty of Science, Taibah University, Yanbu El-Bahr 46423, Saudi Arabia; Manel.dhahri@gmail.com; 5 Nanomedicine Department, Institute for Research and Medical, Consultations (IRMC), Imam Abdulrahman Bin Faisal University (IAU), Dammam 31441, Saudi Arabia; fsalahmari@iau.edu.sa; 6 Department of Medical Sciences and Public Health, Università di Cagliari, Cittadella Universitaria, 09042 Monserrato, Italy

**Keywords:** NMR, drug design, drug development, drug discovery, therapeutics

## Abstract

Studying disease models at the molecular level is vital for drug development in order to improve treatment and prevent a wide range of human pathologies. Microbial infections are still a major challenge because pathogens rapidly and continually evolve developing drug resistance. Cancer cells also change genetically, and current therapeutic techniques may be (or may become) ineffective in many cases. The pathology of many neurological diseases remains an enigma, and the exact etiology and underlying mechanisms are still largely unknown. Viral infections spread and develop much more quickly than does the corresponding research needed to prevent and combat these infections; the present and most relevant outbreak of SARS-CoV-2, which originated in Wuhan, China, illustrates the critical and immediate need to improve drug design and development techniques. Modern day drug discovery is a time-consuming, expensive process. Each new drug takes in excess of 10 years to develop and costs on average more than a billion US dollars. This demonstrates the need of a complete redesign or novel strategies. Nuclear Magnetic Resonance (NMR) has played a critical role in drug discovery ever since its introduction several decades ago. In just three decades, NMR has become a “gold standard” platform technology in medical and pharmacology studies. In this review, we present the major applications of NMR spectroscopy in medical drug discovery and development. The basic concepts, theories, and applications of the most commonly used NMR techniques are presented. We also summarize the advantages and limitations of the primary NMR methods in drug development.

## 1. Introduction

**Scheme 1:**
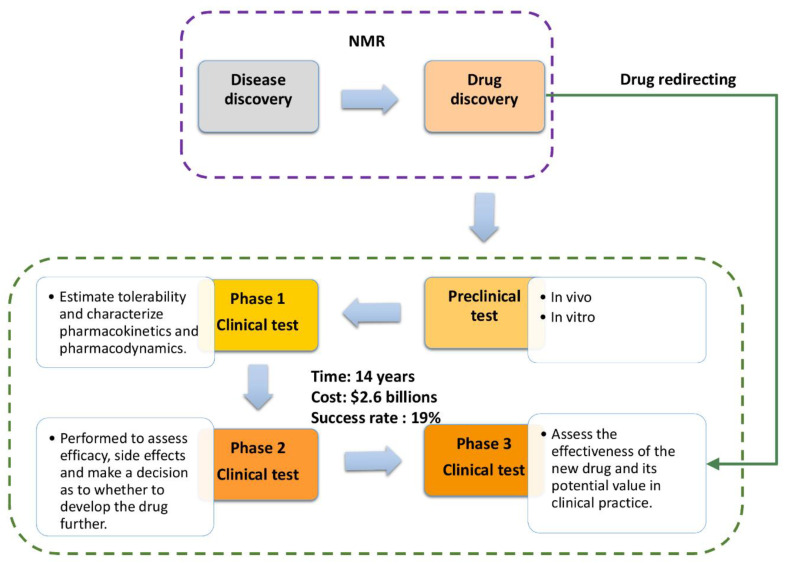
Schematic representation of drug discovery [4]. Numerical data were reported from Meigs et al. [3].

The unexpected SARS-CoV-2/COVID-19 outbreak, with over 34 million confirmed cases globally (Oct. 2020) and the struggle for survival in the absence of a proven and efficient treatments, emphasizes the critical need to develop effective, novel, and rapid drug discovery methodologies. Even though the pharmaceutical industry works constantly to discover and develop novel drugs, the process is still slow and expensive. The cost of introducing a new drug has increased steadily, with current cost estimates predicting that a future drug will cost in excess of $2.6 billion. The typical development cost is usually spread out over the course of 14 years [1,2,3], making investment even more difficult (i.e., cost recovery delay). This high investment barrier for drug development is a result of numerous testing phases (Scheme 1), with each phase requiring a statistically significant number of cases. Although there are several other substantial costs to drug development, that discussion of experimental methods to reduce costs is beyond the scope of this review.

The emergence of a pandemic and the emergencies it creates worldwide understandably drive and motivate the rapid development and/or optimization of drugs. However, patient safety and subsequent earned public trust is a primary requirement. Drug redirecting/repurposing (Scheme 1) is an efficient short-cut method in disease treatment that utilizes existing tools, and combines artificial intelligence, machine learning algorithms, and experimental NMR techniques (i.e., “*from Bench to Bedside*”). This process must be relatively rapid and efficient to have any benefit to patients and the health-care system.

Compared to mass spectrometry and high-performance liquid chromatography (HPLC), nuclear magnetic resonance (NMR) is another powerful technique with several unique advantages [5,6,7,8]. NMR is intrinsically quantitative, and it provides several different approaches that are routinely utilized to identify and structurally elucidate molecules of interest [9,10,11,12,13,14,15,16,17,18]. In contrast to mass spectrometry, NMR is non-destructive, non-invasive, has extremely high reproducibility permitting researchers to acquire measurements under different experimental conditions (e.g., temperature, time points, and concentrations) often while the same sample is inside the magnet [19,20,21,22,23]. NMR can be used in reaction kinetic studies while several consecutive measurements are taken, and while spectral changes (function of the reaction time) are analyzed [24,25,26,27,28]. Moreover, molecules are studied at the atomic level [29,30,31]. Unlike other analytical tools, NMR provides dynamic information, and NMR experiments can be carried out under physiological conditions (e.g., atmospheric pressure, temperature, and different pH values) [32,33]. This is especially important in medical drug design since one must understand the interactions between an enzyme of interest and the ligand(s). NMR provides information on the binding affinity of such ligands, details/location of the binding site, and associated structural changes following binding [32,33,34,35]. These biophysical details are essential when evaluating the potential efficacy of a drug, and during any subsequent optimization. The available literature [32,33,34,36] highlights the practicality of NMR in drug design studies. For these reasons, NMR spectroscopy is highly sought after in drug development [37,38,39,40,41], for both molecule identification [11,13,14,18,42,43,44,45,46] and structural elucidation [15,16,17,45,47,48,49,50,51]. NMR has been successfully applied in stereochemistry [52,53,54,55,56] and isomer determination [57,58,59,60,61], in drug-protein interactions studies [62,63,64], and in the evaluation of drug toxicity [65,66,67,68].

**Figure 1:**
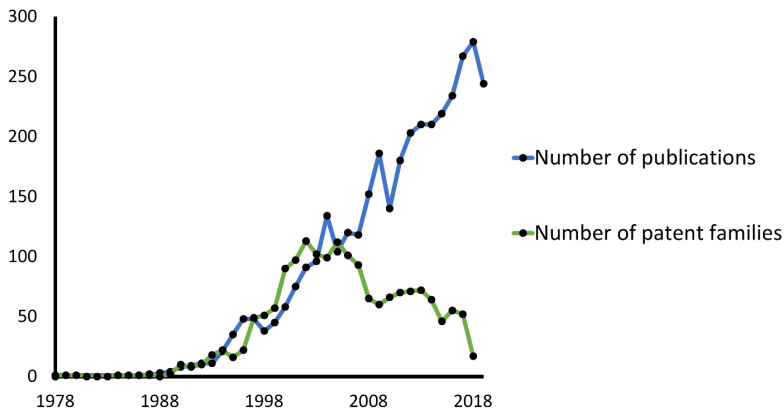
Data search results for: “NMR” and “Drug design” in Scopus (blue) (www.scopus.com) and Orbit patent (orange) (www.orbit.com) databases.

The use of NMR in drug design is not restricted to academic laboratories and gained interest from those in development industries. The use of NMR in drug development increased in the late ‘80s, as seen in both scientific and patent literature (Figure 1). While scientific interest in NMR is still growing, the number of patents has been decreasing since the early 2000s. The top applicants of NMR in pharmaceutical patents are Bristol Myers, AstraZeneca, and Wyeth, with 146, 104, and 67 patent families, respectively.

In addition to the advantages provided by NMR, it is often used with complementary methods such as X-ray crystallography, HPLC, and mass spectrometry [69]. An example of this is found in work by Wyss et al. [36], where they combined X-ray crystallography with NMR fragment-based screening to create the first inhibitor candidate for BACE-1 in Alzheimer’s disease. BACE-1 is a membrane-anchored aspartic acid protease and is responsible for the production of amyloid beta peptides in neurons related to the progression of Alzheimer’s disease [36,70]. Using NMR fragment-based screening, Wyss et al. identified isothiourea as binding to BACE-1 and confirmed this observation with the X-ray crystal structure of the complex of a ligand-efficient isothiourea fragment. Information obtained from these experiments aided in design optimization, resulting in the selection of iminopyrimidinones as BACE-1 inhibitors [36]. This is a perfect example of using different complementary methods to maximize scientific outcome. However, in order to be efficient, one must know the advantages and disadvantages of each method. One of the major issues regarding NMR is the effective size restriction when measuring targets such as proteins above 40kDa. Recent progress has extended this mass limit; an example of this is the resolved structure of chaperone SecB in complex with unstructured proPhoA (PDB ID 5JTL) with a total mass of 119kDa using NMR [71]. In this review, we present practical guideline to use NMR techniques in drug design studies and provide examples of the successful use of NMR in drug-design.

## 2. An Introduction to NMR Spectroscopy

**Figure 2:**
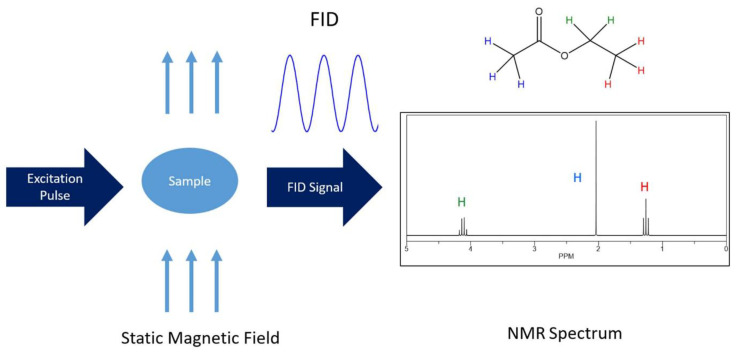
The most simplified layout of an NMR experiment. The NMR spectrum of ethyl acetate Table 1. H chemical shift assignments is shown as an example. The chemical structure and simulated ^1^H-NMR spectrum were created using ChemDraw 18.1.

NMR is a versatile tool for studying biomolecules of all kinds and is a unique regarding the biophysical analysis of drugs [72,73,74,75]. The basic feature of NMR lies in the fact that it inductively detects the Larmor precession of individual nuclei (i.e., spins) which vary because of different atomic, electronic, and chemical environments (i.e., structural atomic relationships). Initially, the sample is placed in a strong, static, and homogeneous magnetic field. Because spins contain angular momentum, they exhibit Larmor precessions around this static magnetic field. A net magnetization builds up over time as the spin population (represented by different energy levels) is minutely differential in the presence of the magnetic field. These levels are dictated by the spin quantum number and can be roughly thought of as different orientations with respect to the static field. Subsequently, induced electromagnetic fields at radiofrequencies (called RF pulses) are applied transverse to the plane of the static magnetic field, and the net or bulk magnetization undergoes an effective rotation. The bulk coherence moves into the transverse plane and the subsequent coherently precessing magnetization vectors induce a detectable alternating voltage in the NMR receiver coil. This tiny alternating voltage is amplified and converted from an analog time domain signal to a frequency reading via Fourier transformation. These signals are recorded in response to the induced radio-wave pulses (Figure 2) and are representative of the Larmor frequencies that are converted into normalized values termed chemical shifts in order to be field independent. 

This is the final, representative spectroscopic signature of the chemical and magnetic environment of the atom, and it provides detailed atomic resolution information about the molecular structure [76,77,78,79]. A wealth of information can be derived from the NMR signal made up components such as the chemical shift position, signal linewidth, and observed couplings/multiplet structure. The signal contains precise details about the chemical environment of the involved and interacting spins in the structure of the molecule, dynamics of the spins in various timescales, conformational exchange, etc. [80,81,82]. Any change in the environment of the associated spin can be observed. These changes include molecular binding, interactions, and/or exchange between different conformations [20,83,84,85]. Thus, NMR has been used to study a wide range of functional molecules such as natural products [86,87,88], saccharides [89,90], metabolites [91,92], DNA [93,94], and proteins [95], and its use as an analytical tool in drug design research has increased immensely in recent years (see Figure 1).

As NMR is non-destructive in nature, the same sample can be analyzed repeatedly. NMR can be performed first and then submitted to mass spectrometry (MS); however, the addition of common deuterated NMR solvents (such as D_2_O) can perturb MS results and should be avoided (e.g., tube-in-tube or by using non-deuterated solvent and running the NMR unlocked). In fact, high-performance liquid chromatography (HPLC), ion-trap MS and NMR have been combined to detect the effects of drugs demonstration in urine and blood serum samples [69,96,97]. Corcoran and Spraul [98] emphasize that liquid chromatography (LC), MS, and NMR utilized in parallel give comprehensive structural data on molecules of novel drugs in development.

In the following subsections we briefly describe NMR methods that have been used in drug design, and then discuss how NMR principles are used in drug discovery research.

### 2.1. One Dimensional NMR Spectroscopy

The one-dimensional (1D) experiment is by far the most common NMR experiment used for drug studies. The 1D acquisition takes the least amount of time, has one of the simplest hardware requirements, and therefore, in most cases, 1D-NMR is more attractive for high throughput studies. One dimensional NMR spectroscopy normally incorporates a preparation period, some form of induced excitation to form coherence, and lastly, a signal “read” detection period. The preparation period can be modified according to the needs of the experiment or the specifics of the sample. Simple 1D NMR is capable of rapidly producing high-quality spectra of drugs and their targets while revealing how the drugs and targets may interact at the atomic level. 1D ^1^H-NMR is extremely effective in drug design studies because it has a (relatively) high sensitivity, it is non-destructive, and because hydrogen atoms are extremely abundant in most molecules of interest. Therefore the resulting spectra usually contains a large amount of relevant information and this wealth of data can be acquired in a relatively short period of time. The basic 1D ^1^H-NMR, along with 1D ^13^C-NMR, 1D ^15^N-NMR, and 1D ^31^P-NMR, and their respective uses in drug design/discovery are briefly discussed below.

#### 2.1.1. 1D ^1^H-NMR

The ^1^H hydrogen isotope is NMR visible, has the highest gyromagnetic ratio (apart from tritium) of all of NMR active nuclei, and is combined with a vast natural abundance in organic chemical compounds. This makes the 1D-^1^H-NMR experiment the most commonly applied NMR approach. Moreover, many software databases [99,100,101,102] are well established for ^1^H-NMR spectra therefore assisting with processing, analyzing, and identifying the detected molecules automatically. Since almost all drug discovery and drug development studies are performed on samples dissolved in water, many different solvent suppression methods have been applied. The most common is presaturation [103,104]. The key point of this method is to use a low power induced field at the specific frequency of water. This effectively averages out any coherence of the water resonance. The experiment is simple for common hardware to perform and easy to set up; however, presaturation has a substantial disadvantage in that signals resonating close to the solvent signal will show decreased intensity [103,104] or may be lost entirely. This is due to the fact the even selective pulses or very low power pulses also excite some area around the water signal. Also suppressed hydrogen from H_2_O in solution can exchange with atoms of interest in the molecule and effectively bleed the suppressive spin state to any neighboring atoms. The water signal itself is usually broad, so a wider area of suppression is not necessarily undesirable but affects more of the molecule(s) of interest. More recent water suppression techniques have been developed such as those based on a scheme known as excitation sculpting [105,106]. The basic pulse sequence consists of a double pulsed field gradient echo (DPFGE) in each of which a selective component pulse is flanked by two pulsed field gradients [107]. The particular elements differ for different applications. In the case of water suppression known as WATER suppression by GrAdient Tailored Excitation (WATERGATE), this involves an initial encoding gradient along with the middle element; a combination of two selective 90° rotations on the water along with a central non-selective 180° excitation of all resonances [108]. This is predicated in that water experiences a 360° rotation (effectively nothing) while all other spins experience 180° rotation. The application of the second refocusing gradient does not rephase the water and therefore removes the signal. The reader is referred to the detailed literature [103,104,109] for further information. In principle, a water suppression element (or many elements combined) can be incorporated in any existing pulse sequence to enhance the performance, and it has been implemented in various 1D, 2D, and triple resonance 3D/4D experiments. Although ^1^H is the most sensitive nucleus for NMR yielding strong, sharp signals within a few minutes [110], chemical shift dispersion of ^1^H is quite narrow (only around 10 ppm). This has prompted the consideration of other nuclei such as ^13^C, ^15^N, or ^31^P for resolution improvements.

#### 2.1.2. 1D ^13^C-NMR

Compared with ^1^H, ^13^C has a much higher chemical shift dispersion (~200 ppm), however the natural abundance of ^13^C is low (1.1%). Additionally, the gyromagnetic ratio is ~4 times weaker than ^1^H and therefore ^13^C spectra are far more difficult to obtain especially for less concentrated samples. There are some polarization transfer techniques such as Distortionless Enhancement by Polarization Transfer (DEPT) or Insensitive Nuclei Enhanced by Polarization Transfer (INEPT), which can enhance signal intensity by starting the magnetization on a higher sensitivity and abundance proton and then transferring magnetization to the less sensitive carbon nuclei for subsequent direct detection [111], but this requires additional hardware and acquisition times. The use of 1D ^13^C in drug design studies was illustrated by Tsujimoto et al. [112]. The goal of the study was to examine if a metabolomics approach based on ^1^H and ^13^C offers significant improvements when comparing potential drugs. The authors prepared a total of 40 samples with five different citrus-type crude drugs (kijitsu, tohi, chimpi, kippi and seihi) and measured 1D ^1^H and 1D ^13^C for each sample. While ^1^H-NMR spectra allowed the identification of three compounds (naringin, sucrose, and β-glucose), using ^13^C-NMR allowed unambiguous identification of eight additional compounds (naringin, neohesperidin, α- and β-glucose, sucrose, limonene, narirutin, and synephrine). The added signal resolution from ^13^C-NMR spectra allowed researchers to obtain better structural information about the compounds than from ^1^H-NMR spectra alone.

#### 2.1.3. 1D ^15^N-NMR

In comparison to the previous example, ^15^N has a lower shift dispersion (~100ppm) than ^13^C, but higher than that of ^1^H. Here, the situation is unfortunately severely limited due to an even lower natural abundance (0.37%) and a gyromagnetic ratio ~10 times smaller than ^1^H. This means that ^15^N’s combined sensitivity is around 260,000 times lower than ^1^H. As a result, isotopic enrichment of ^15^N combined with ^1^H-mediated enhancement via indirect detection is often needed in order to obtain a satisfactory 1D ^15^N spectra. Similar to ^13^C, a few methods are available to overcome such low sensitivity. One of them focuses on tagging molecules with carboxyl groups using ^15^N-ethanolamine and later detecting the signal using a 2D heteronuclear correlation NMR experiment [113]. Currently, novel approaches such as “smart isotope labeling” have been developed [114]. Also, the SOFAST (Band-Selective Optimized Flip Angle Short Transient) technique can help but results in substantial hardware considerations/drawbacks and often increased concentrations, and/or dramatically longer experiments are still required [115,116,117].

Promising methods are on the horizon. These methods include ^15^N heteronuclear signal enhancement via Signal Amplification by Reversible Exchange in SHield Enables Alignment Transfer to Heteronuclei (SABRE-SHEATH); however, more work and research are required before such methods can be applied for biomedical purposes [118].

#### 2.1.4. 1D ^31^P-NMR

With a natural abundance of 100% and a gyromagnetic ratio of about 2.5 times smaller than ^1^H, one may think that phosphorus could be broadly used for NMR experiments regarding the drug discovery and development. However, the application of ^31^P is limited due to the fact that most of the molecules of interest simply do not contain a phosphorus atom. Therefore ^31^P-NMR is usually applicable for studies related to energy, phospholipid metabolism (ATP, NADP), and/or characterization of changes in DNA [94,119,120]. For example, Overall et al. conducted an experiment in which they showed that ^31^P solid-state NMR can be used for quantitative analysis of DNA dynamics within live bacteria [94]. For that, the researchers first prepared untreated cultures of *E. coli*, and measured them using a Hartmann-Hahn ^1^H to ^31^P cross-polarization (^31^P CP) experiment. Afterwards, they measured *E. coli* treated with ampicillin and maculatin 1.1 (Mac1.1) in a similar manner. Spectra obtained from treated bacteria compared to those obtained from untreated bacteria showed alterations in the lineshape, reduced signal intensity at the spectrum’s edges, and a shift in spectral density towards 0 ppm which indicated the increased dynamics of the phosphorus from nucleic acids [94].

Over time, several innovations have been applied to expand the usage of ^31^P. Like in ^13^C and ^15^N labeling of specific biological compounds, incorporation of ^31^P can also be used. In order to achieve that, 2-chloro-4,4,5,5-tetramethyldioxaphospholane (CTMDP) can be used for tagging lipids containing hydroxyl, aldehyde, and carboxyl groups that can later be detected with better resolution [121]. Another fairly recent method enables toxicological screening of ^31^P in living cells for several hours without affecting cell viability [122]. This specific method can be used to observe the changes in energy metabolism in real-time while enabling the evaluation of the effects of administered drugs.

### 2.2. Multi-Dimensional NMR Spectroscopy

NMR experiments are not limited to one-dimensional direct acquisition; they can be extended to multidimensional methods including 2D, 3D, 4D, and even higher dimensionality. The focus of this section is common 2D NMR experiments that have been used in drug design and drug development. A brief description of Correlation Spectroscopy (COSY), Total Correlation Spectroscopy (TOCSY), and Heteronuclear Multiple Bond Correlation (HMBC), along with their uses in drug design and discovery will be presented.

#### 2.2.1. 2D ^1^H,^1^H-COSY

COSY is one of the simplest and most frequently used 2D NMR experiment [123]. It shows the homonuclear coupling of nuclei (i.e., ^1^H-^1^H) separated by up to several covalent bonds. The pulse sequence consists of a 90° excitation pulse followed by a specific evolution time (t_1_), a second pulse, and finally a measurement period (t_2,_ not to be confused with relaxation rates or times). The second pulse can be 90° or 45° or 135°, depending upon the specific requirements, and respectively yield COSY [124], COSY-45 or COSY-135 functionality (see [125,126,127]). A two-dimensional Fourier Transform (FT) yields the final spectra and shows the frequencies for proton (^1^H) or carbon (in the case of carbon detection) along both axes. There are two types of peaks; (I) Diagonal peaks, which represent the peaks of the conventional 1D spectra, and (II) cross-peaks, which have different values in the two frequency axes and are therefore off the diagonal. These off diagonal cross-peaks are the most important pieces of information as they mark correlations between pairs of nuclei due to through bond magnetization transfer. This helps in identifying which atoms are connected [128], critical for structural elucidation of both known molecules and unknown molecules in solution [129]. By implementing phase-cycling [130,131], it is also possible to distinguish different types of coupling and yields further helpful information about the chemical structure of a molecule [132]. As an example, the use of the COSY experiment was presented in the work of Zheng et al. [88]. The main goal of their work was to investigate potential biological differences and compare the pharmacological effects between Danggui (an herbal drug used in traditional Chinese medicine) and European Danggui. For that, Zheng et al. treated blood deficiency rats with Danggui and European Danggui and collected samples of their serum and urine. The samples were later measured using ^1^H-CPMG-NMR, ^1^H-NOESYPRESAT-1D, ^1^H,^1^H-COSY, and ^1^H,^13^C-HSQC, and then compared to equivalent spectra from untreated rats. The results showed that exposure to Danggui and European Danggui altered the levels of 18 different metabolites, such as lactate, nicotinamide, glycerol and formate, which were involved in a total of seven different metabolism pathways. Additionally, it was proven that Danggui and European Danggui have different chemical compositions, with Danggui having better blood-enriching effects than European Danggui.

#### 2.2.2. 2D ^1^H,^1^H-TOCSY

Total Correlation Spectroscopy (TOCSY) also originally known as the Homonuclear Hartmann Hahn (HOHAHA) experiment can be considered an extension of the 2D ^1^H,^1^H-COSY experiment. The difference between the two experiments is that a TOCSY experiment will show multiple cross-peaks including indirectly coupled nuclei (i.e., longer range via scalar coupling) throughout the J-coupled spin system of a chemical compound. The basic pulse sequence of the TOCSY consists of excitation by a 90° pulse, followed by a free variable evolution period which encodes the indirect dimension. This is normally followed by an isotropic mixing sequence to transfer magnetization between spins via the strong scalar coupling. The mixing generates in-phase magnetization throughout a spin coupled network of the associated nuclei during the mixing time. Lastly, a direct detection is performed. A major advantage of the TOCSY experiment is that it detects in-phase magnetization (i.e., pure absorptive line-shape) which is far easier to analyze compared to the anti-phase information in the phase sensitivity COSY-type experiment. The isotropic mixing is usually performed using a composite pulse scheme such as WALTZ, MLEV or DIPSI [133,134] pulse train, and can be sandwiched between two z-filters [135] where isotropic mixing is performed on the longitudinal magnetization. The most obvious advantage of TOCSY is that all cross-peaks of the same spin system can be observed for whole spin system at once. This is useful for identifying the complete network of spins and reducing the ambiguity of any spectral overlap. The TOCSY experiment can be produced as 1D with a relatively shorter time and easier analysis compared to 2D but lacks the benefit of multi-dimensional resolution. The 2D TOCSY is usually done to resolve spectra overlap [50] when first identifying molecules [136,137,138]. For example, Jiang et al. used this to predict the response to gemcitabine-carboplatin (GC) chemotherapy in patients with metastatic breast cancer who were previously exposed to treatment with both anthracyclines and taxanes [137]. For that, researchers collected serum samples from 29 patients prior to treatment and measured them using 1D ^1^H-NMR. Additionally, they conducted 2D NMR experiments such as the ^1^H,^1^H-COSY, ^1^,^1^H-TOCSY, ^1^H,^13^C-HSQC, and ^1^H,^13^C-HMBC to help assign serum metabolites. After receiving the treatment with gemcitabine-carboplatin, patients were divided into four groups based on the results from the computed tomography: complete response (CR), partial response (PR), stable disease (SD), or progressive disease (PD). After comparing NMR results prior to the treatment with the outcome of chemotherapy, the researchers observed lower baseline levels of serum format and acetate in breast cancer patients who progressed with the disease than in those who achieved a clinical benefit from therapy, indicating that those two biomarkers could be used to distinguish between patients who will benefit from GC treatment from those who do not [137].

#### 2.2.3. 2D ^1^H,^13^C-HSQC

**Figure 3:**
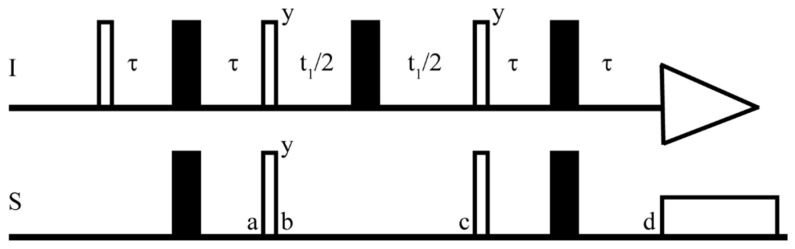
The pulse sequence of HSQC. I and S represent two heteronuclear spins. The τ is (1/4J) where J is the coupling constant. The thick and thin bars represent the 180° and 90° pulse respectively [142].

2D- Heteronuclear Single Quantum Coherence (HSQC) experiments are commonly used to help resolve spectral overlap [139] while providing ^13^C information without the inherent sensitivity losses involved in ^13^C direct detection (see below). HSQC shows the correlations between directly coupled nuclei [140], e.g., ^1^H-^13^C or ^1^H-^15^N [140]. As such, an HSQC spectrum will show clean peaks for each unique proton directly connected to the heteronuclear nuclear atom of interest [140,141]. In ^1^H,^13^C/^15^N HSQC experiments, the magnetization is transferred from the more sensitive nucleus (I:^1^H) to the less sensitive nucleus (S:^13^C/^15^N) [142,143,144] (Figure 3). This is especially useful when applying NMR spectroscopy to drug design, as most drugs are organic (i.e., contain carbon atoms), and the relative abundance of ^13^C (1.1%) is quite low [143]. By transferring sensitivity from ^1^H to ^13^C, one can circumvent the long experimental time required for 1D ^13^C experiments [143].

For example, De Castro et al. [145] studied Ptac2S and its related cytotoxicity to the cisplatin-resistant epithelial ovarian carcinoma (EOC), Skov-3 cells. In the study, they used NMR spectroscopy and multi-variate statistical analysis to observe how Skov-3 cells reacted to treatment with Ptac2S. In particular, they used ^1^H,^13^C-HSQC along with ^1^H-COSY and Heteronuclear Multiple Bond Correlation (HMBC), and the Human Metabolome Database to assign the chemical shifts of the lipid metabolites present in the studied samples. Interestingly, Skov-3 cells treated with Ptac2S produced more pyruvate than Skov-3 cells treated with cisplatin. The authors also noticed an unexpected difference in lipid metabolite expression levels between the cells treated with Ptac2S and those treated with cisplatin. These results provide a possible explanation for how Ptac2S is able to overcome cisplatin resistance in Skov-3 cells [145].

#### 2.2.4. 2D ^1^H, 13C-HMBC

Heteronuclear 2D experiments are useful for transferring magnetization from sensitive nuclei (i.e., ^1^H) to less sensitive nuclei (i.e., ^13^C) [146] thereby reducing the time needed for the acquisition of spectra [147]. Heteronuclear Single Quantum Coherence (HSQC) will only show one cross peak for each coupled pair [92,128] of nuclei. This makes HSQCs useful for assigning the backbone of proteins [148] and in metabolites of complex biofluids [149], whose 1D ^1^H-NMR spectra can suffer from severe spectral overlap.

The HMBC technique, while similar to HSQC, is an example of a heteronuclear 2D experiment that reveals correlations between nuclei separated by two or more chemical bonds while also suppressing one-bond correlations at the same time. This experiment combined with HSQC is often used to assign NMR spectra for studied molecules in drug design experiments [65,66,137,145].

As an example, HMBC was used in a recent study by Xu et al. [66] to investigate the changes in the metabolic profiles of rats treated with different dosages of the “RenqingMangjue” pill, a traditional Tibetan medicine. In this study, the rats were divided into four groups based on the amount of “RenqingMangjue” administered: low dose group (LD)-83.33 mg/kg/day, middle dose group (MD)-333.33 mg/kg/day, high dose group (HD)-1333.33 mg/kg/day and a control group (NC). After 15 days of consecutive administration, half of the rat population was used to collect samples such as serum, kidney, and liver tissue, while the other half underwent an additional 15 days of recovery before the same samples were acquired. The samples were measured using ^1^H-NMR CPMG (an experiment used to suppress signals from larger molecules, see below) [150,151,152] along with ^1^H,^1^H-COSY, ^1^H,^13^C-HSQC, and ^1^H,^13^C-HMBC used for molecules assignment. The obtained spectra showed that the “RenqingMangjue” pill alters many metabolites, which are related to a variety of metabolic pathways including energy metabolism, amino acid metabolism, and lipid metabolism indicating potentially harmful effects on kidneys and liver.

#### 2.2.5. Relaxation-Edited NMR Spectroscopy

**Figure 4:**
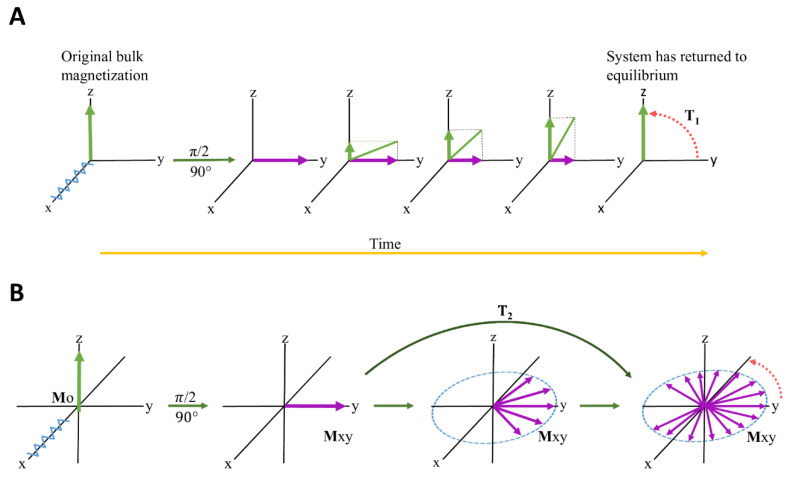
RF (radiofrequency) pulse causes the bulk magnetization to move to the transverse plane. Over time (ms to seconds, and in extreme cases, minutes [155]), the bulk magnetization will decrease in the transverse plane, and increase in the longitudinal axis, returning to its original, equilibrium value. (**A**) represents T_1_ relaxation and (**B**) represents T_2_ relaxation.

Relaxation in NMR is a phenomenon describing the time dependence involved in signal intensity after an induced RF (radiofrequency) pulse is applied [153]. After application of a 90° RF pulse, the bulk magnetization will move to the transverse (xy) plane and will gradually return to its original equilibrium position along the longitudinal (z) axis [154]. This process is described in Figure 4, and is termed T_1_ relaxation. The details are beyond the scope of the manuscript and interested readers are directed to [155] and references therein. Relaxation times for NMR are even more complicated and exist in two categories: T_1_ and T_2_. T_1_ refers to the rate of longitudinal (or spin-lattice) *Z*-axis relaxation as the system returns to equilibrium. A second component also contributes, i.e., T_2_ relaxation and refers to the rate of transverse (or spin-spin) relaxation [154] which occurs in the XY plane. T_2_ is independent of the longitudinal relaxation (T_1_) and represents the loss of coherence in the precessing spins. Therefore NMR relaxation spectroscopy can be based on T_1_ and/or T_2_ [156], and is collectively referred to as “relaxation edited NMR” [157].

T_1_-based methods typically measure and compare the T_1_ times of the free and bound ligands. A common way to measure the T_1_ value of a small molecule is the inversion recovery experiment [158,159], although other experiments are also available such as ultrafast NMR T_1_ [160] and saturation inversion recovery [161]. In general, the shorter T_1_ the relaxation time the less intense the peak signal will be and the broader the signal linewidth [162]. The T_1_ values of free and bound ligand will differ depending on how strongly the ligand binds because molecular interactions with the target will influence the ligand’s molecular motion, and hence, its longitudinal relaxation [156]. Bound ligands will have smaller T_1_ values than in their free form because, overall, they will experience slower molecular motion upon interacting with a target [163] therefore behaving like a much larger molecule. They can (depending on molecule size) also display a negative NOE difference spectrum (transferred NOE) [164], whereas non-binding ligands normally show small-positive NOEs [156]. For binding ligands to display negative NOEs, their T_1_ values must be comparatively longer than the 1/k_off_ value of the target [156].

T_1_ relaxation times can be easily used to screen small molecules as ligands for DNA [165] and serve as a basis for HTS experiments [166]. An experiment related to drug design that utilized 1D and 2D relaxation edited NMR was done by Hajduk et al. [167] in which he and others used 1D and 2D relaxation edited NMR techniques to detect ligands that bind to FK506 binding protein and stromelysin. One year earlier, Liu et al. [157] used relaxation edited one-and two-dimensional ^1^H-NMR spectroscopy to characterize biological fluids. Tang et al. [168] extended this by applying relaxation edited NMR Spectroscopy to improve the detection of metabolites in blood plasma. More recently, Jaremko et al. commented on available models used to interpret ^15^N protein relaxation data [169], and even used deficient ^15^N relaxation data to rapidly calculate the dynamics of proteins [170].

**Figure 5:**
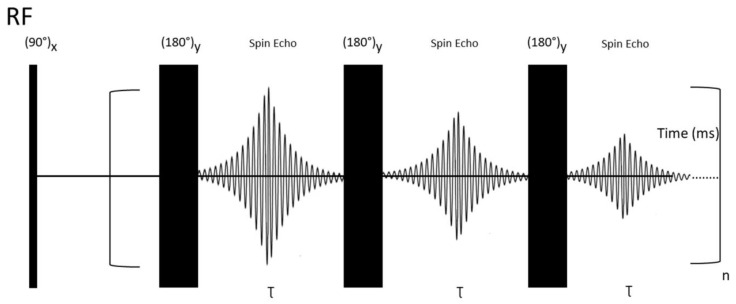
CPMG pulse sequence. First, a 90° RF pulse is applied and results in transverse magnetization in the xy plane. Then a 180°_y_ pulse is applied to re-phase the magnetization vectors. After 180°_y_, the vectors who were faster during the dephasing are overtaken by the slower vectors, which results in re-phasing and generation of a spin-echo signal. This process is repeated several times.

The T_2_ relaxation experiment relies on so-called Carr–Purcell–Meiboom–Gill (CPMG) building blocks (Figure 5).

This pulse sequence is explained with the following steps: First, application of a 90° RF pulse creates a transverse (xy plane) magnetization. Second, a spin-echo period (delay-180°-delay block) is responsible for Mx/y magnetization decay. This period is repeated “n’’ times (CPMG building blocks). It is essential to point out that every NMR experiment involving a large number of pulses (e. g. due to the repeating building blocks) is likely to be sensitive to hardware restrictions and small miscalibrations of the duration of the applied pulses. To attenuate the unwanted effects of miscalibrations, Meiboom and Gill modified the previously used Carr–Purcell sequence [171] by changing the phase of the applied 180° pulses from x to y [172]. This procedure can be used to measure T_2_ relaxation times of any type of nuclei. For instance, in the case of ^13^C, all pulses and acquisitions are applied on ^13^C channel, while broadband proton decoupling is applied during all pulse sequences. It works analogically for different NMR-active nuclei [173].

In a typical CPMG experiment, the effective transverse relaxation rate, R_2,eff_, is typically measured by fitting the signal decay as a function of a variable number of CPMG blocks [174]. The experimental half-height linewidth (d) of a given resonance signal is directly related to T_2_^*^ (also called as ‘effective’ or “observed’’) by the following equation:

(1) $$d=\frac{1}{\pi \;T_{2}^{*}}$$

T_2_ represents the transverse relaxation times, and additional broadening comes from the magnetic field inhomogeneities (T_2_^inh^), which must be taken into account.

(2) $$\frac{1}{T_{2}^{*}}=\frac{1}{T_{2}}+\frac{1}{T_{2}^{inh}}$$

T_2_ measurements of ligands are also useful for determining the binding nature of a small molecule. The T_2_ values of small molecules are quite large compared to those of bigger molecules (i.e., proteins) mostly because macromolecules have more spin-spin diffusion [175]. Bound ligands will, therefore, display shorter T_2_ values than non-binding ligands because they interact with the target (i.e., protein), adopting similar vibrational and rotational energies to the target [176]. This interaction is represented by the resonance line broadening in the binding ligand’s spectrum when a receptor is introduced into the sample [156]. Given the sizable difference of T_2_ values of binding and non-binding ligands, one can utilize 1D relaxation-edited experiments to distinguish the binding ligands from the non-binding ligands efficiently and effectively based on the differences in the T_2_ values [167]. These and other related relaxation edited experiments prove useful in drug design.

Relaxation edited NMR spectroscopy takes advantage of an inherent atomic property (i.e., the return of bulk magnetization back to equilibrium [177]), so no molecular enrichment (e.g., ^15^N isotopic enrichment of protein targets) is required [167]. Furthermore, the slow time scale of NMR relaxation allows the user to manipulate the external conditions (i.e., length and power of pulse) to increase the resolution of targets and potential drugs [155] in NMR drug design experiments. However, this slow timescale also sets the lower limit at which NMR drug design experiments can be performed [155], meaning that any external manipulations cannot decrease experimental time below a certain threshold. This varies based on the drugs and targets used in the experiment. Low drug solubility is also a challenge, as the ligands must be at a sufficiently high concentration to allow detection via NMR, although the use of organic solvents has helped to attenuate this effect in relaxation edited NMR spectroscopy [156]. For examples of experiments that use different NMR techniques mentioned above, see Table 1.

## 3. NMR Methods for Drug Discovery and Drug Development

As stated, NMR spectroscopy can be fundamental in studying how drugs interact with their targets. This has been done mainly via the Fragment Based Drug Design (FBDD) approach, which has two sub-approaches: target- (i.e., protein) based, or ligand- (drug) based. Target based screening monitors how the target responds to binding molecules in a method called Structure Activity Relationship (“SAR”) by NMR. Ligand (drug)-based screening methods provide ways to observe the binding/non-binding behavior of the drug in approaches such as Saturation Transfer Difference (STD) and other Nuclear Overhauser Effect (NOE) type methods, diffusion-based methods, relaxation-based methods (i.e., T_1_ and T_2_). Target based screening, ligand (drug) based screening, and their respective methods, are discussed in detail below.

### 3.1. NMR in Fragment Based Drug Design (FBDD)

NMR-based drug discovery can be broadly classified into two groups: chemical and biological (in-cell) categories. One of the principal methods of drug discovery using NMR spectroscopy is called fragment-based drug design (FBDD) [194]. In-cell NMR (biological) based drug discovery techniques will be discussed later in this review.

**Figure 6:**
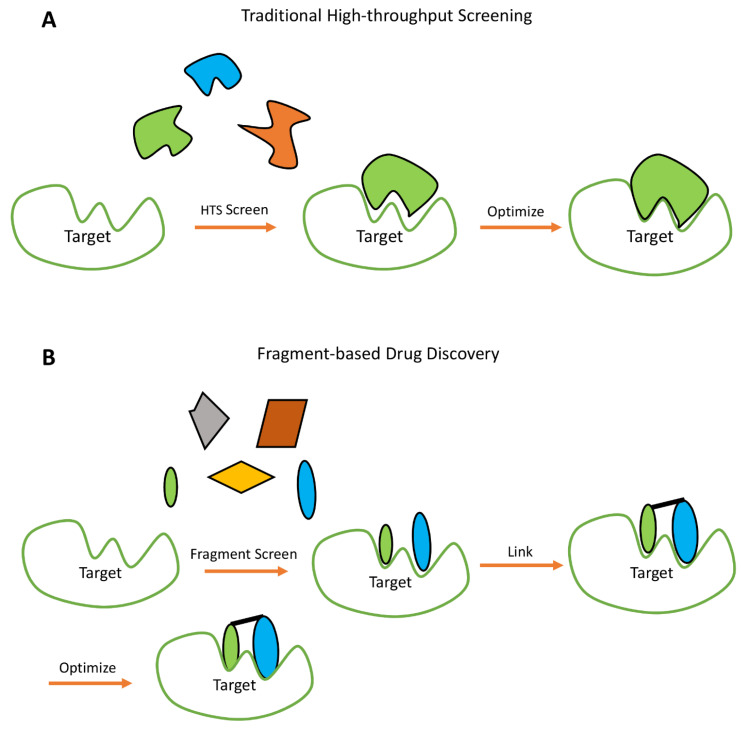
Underlying mechanisms of (**A**) Traditional High-throughput Screening, and (**B**) Fragment Based Drug Discovery.

FBDD was first reported in 1996 [195] and used throughout the late 1990s as evidenced by the use of keywords related to FBDD in papers published during this time [196]. The use of FBDD as a viable drug screening technique began to be widely adopted in the mid-2000s [197]. High Throughput Screening (HTS) is another technique widely used in drug discovery [198]. HTS analyzes molecules from a chemical library to see which ones are suitable leads [198,199,200,201] (see Figure 6). FBDD techniques will screen against a carefully designed fragment library composed of a few thousand molecules (for details on the choice of compounds and design of fragment libraries, see [202,203]) and identified hits are further developed via fragment growing, fragment merging, or fragment linking [194]. For examples of drugs derived from the FBDD approach that are currently in clinical trials, refer to Table 2.

HTS has been productive in drug design [204,205], but the method is time and resource intensive [206] and expensive [206] because of the numerous molecules to be examined (~100 million) [207]. Furthermore, the success rate is only estimated to be at ~50% [204,208]. Unlike traditional HTS, which can survey a large number of molecules ranging from a few hundred thousand to a few million [209], FBDD usually surveys a few thousand molecules (~1000–15000) from libraries with greater chemical diversity [209,210]. FBDD is a main-stream screening technique for drug discovery [207,209,211,212,213,214,215,216] and NMR is standard for many FBDD studies [209]. Additional methods and techniques such as SPR, X-ray crystallography [209,217,218,219,220] etc. have also been used in FBDD studies, accompanied or unaccompanied by NMR experiments. For examples of FBDD derived drugs using methods besides NMR, refer to Table 2.

At the time of writing, and to the best of our knowledge, there are three Food and Drug Administration (FDA)-approved drugs derived from the FBDD approach [221], and over 30 are in clinical trials [222]. The first marketed drug derived via the FBDD approach is vemurafenib [223]. Vemurafenib is also the first drug approved for treatment of *BRAF*-mutant cancer [224], and is reported to exhibit significant clinical benefit for patients with metastatic melanoma [224]. Venetoclax, a common drug used to treat patients with chronic lymphocytic leukemia [225], is considered the second drug to be discovered using the FBDD approach [221], and ribociclib, a CDK4 inhibitor, the third [221]. The names, structures, targets/applications, and clinical status of vemurafenib, venetoclax, ribociclib, and other drugs are listed in Table 2.

As mentioned, NMR spectroscopy can be used in FBDD in two different ways: (1) target (or receptor) based screening, and (2) ligand-based screening. With the stated advantages and disadvantages, researchers must select based on their available compounds.

#### 3.1.1. Target Based Screening

**Figure 7:**
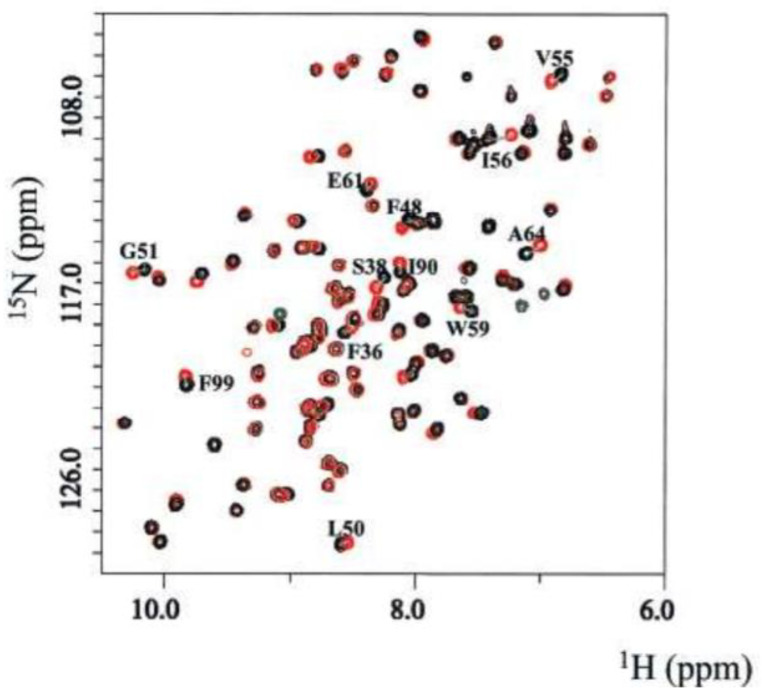
Overlaid 1H-15N-HSQC for FKBP in the absence (black contours) and presence (red contours) of 2-phenylimidazole. Adapted with permission from Hajduk et al. [249].

Target based screening typically utilizes the “SAR by NMR” (structure-activity-relationship by nuclear magnetic resonance) approach [246]. SAR is primarily used to identify and develop extremely tight-binding ligands [247]. The ligand to target binding is traditionally monitored via chemical shift changes [247] using a correlation spectroscopy such as ^1^H-^15^N HSQC starting with the target and no ligand present [248]. Multiple spectra for the target are recorded in the presence and absence of ligands. The binding ligand will cause chemical shift perturbations in the target, and these perturbations are often easily visualized by overlaying the two spectra [247]. For example Hajduk et al. investigated the binding interactions of 2-phenylimidazole with the FKBP protein as shown in Figure 7 [249].

From the overlaid spectra, chemical shift changes are measured, and from the molecular location, extent, and rate of the chemical shift changes, the binding site and affinity of the ligand is calculated [250]. Then, by following a procedure completely analogous to that of FBDD (see Figure 6), a ligand developed from multiple fragments can be optimized for the binding site of interest, again by monitoring the changes in chemical shifts of the target. Several examples of the successful applications of SAR by NMR in drug design research are replete in the scientific literature [204,251,252].

SAR by NMR spectroscopy allows researchers to observe directly ligand binding [247] in both solution state and solid-state spectra [253], increasing the method’s versatility [254]. It works particularly well for targeting proteins with adjacent “subpocket” binding sites [248]. Furthermore, SAR by NMR is cost-effective when combined with HTS (High Throughput Screening) [255]. SAR by NMR can also be used even when atomic peak assignments in spectra are unknown, though it is much more powerful when the resonance frequency of each atom is known [254]. The main limitation of SAR by NMR, however, is its inability to distinguish between multiple binding modes (i.e., cleavage of covalent bonds or allosteric changes), and if multiple binding modes are present, it can be difficult to pinpoint the “true” binding site of the ligand solely using data obtained using SAR by NMR [254].

#### 3.1.2. NMR Ligand-Based Screening

Ligand-based screening, the second approach of NMR in FBDD, has three main categories: 1) Saturation Transfer Difference (STD) and Nuclear Overhauser Effect (NOE) type methods, based on 2) diffusion methods, or 3) relaxation-based methods (i.e., T_1_ and T_2_).

#### 3.1.3. Saturation Transfer Difference (STD)

**Figure 8:**
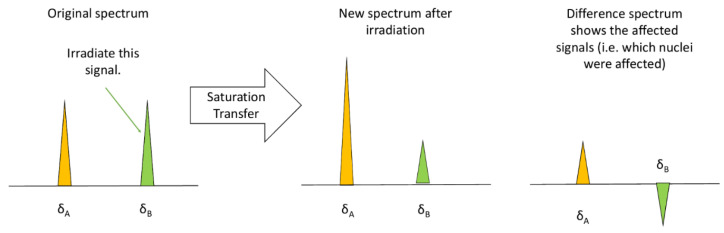
Basic layout of the different NOE effect on NMR signals.

Saturation Transfer Difference (STD) NMR depends on the Nuclear Overhauser Effect (NOE), which is often used to enhance the sensitivity of less sensitive nuclei such as ^13^C and ^15^N [256,257]. This increase in sensitivity is possible because of dipolar coupling (i.e., through space interactions of separate nuclei) [257]. The increase in sensitivity is actually brought about by applying a long, low power radiofrequency pulse that selectively saturates the magnetization [256] of a specific chemical group (i.e., the methyl groups on a protein), which is then given time to transfer to another chemical group via the NOE dipolar coupling within a few angstroms [258]. The transfer in magnetization is easily visualized on a NMR spectrum that takes the differences in the signal intensities from before and after the irradiation. This new spectrum is called a “difference spectrum”, and it reveals what chemical groups interact with the irradiated signal [259] (see Figure 8).

**Figure 9:**
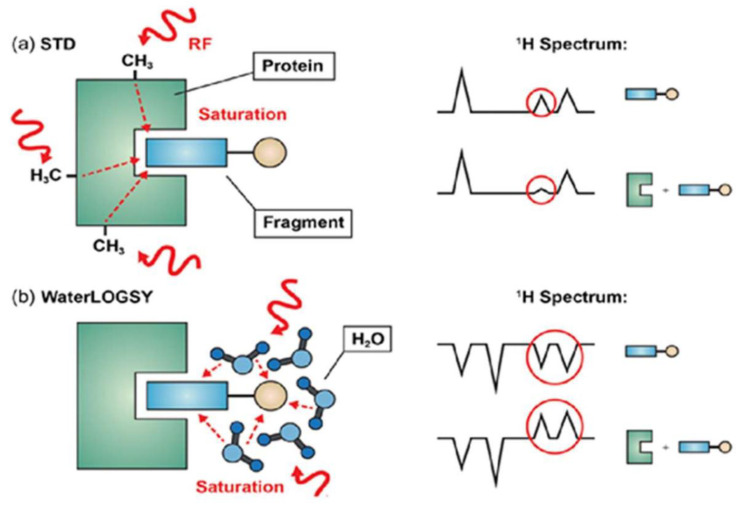
Illustration of STD NMR (**a**) and WaterLOGSY (**b**) in drug design. Copied with permission from Robson-Tull [209].

STD NMR is an application of NOE used to probe the binding of ligands to a specific site within the targeted proteins [256]. A generic example of detecting ligand binding via STD is presented in Figure 9a. The STD NMR method follows the same concepts as a normal NOE experiment: a spectrum of the ligand in the free, non-binding form is recorded, the ligand is allowed to bind to the protein, which has a functional group of interest (i.e., methyls) with a saturated signal from a previous selective radiofrequency pulse. The saturated signal travels to the ligand, increasing the intensity of a signal on the ligand spectrum and finally a difference spectrum is used to determine precisely which sections of the ligands bind. The difference in peak intensities proves the presence of ligand binding [260].

Water-Ligand Observed through Gradient Spectroscopy (WaterLOGSY) is a second type of STD (see Figure 9b). The main difference with normal STD NMR is that water is the saturated signal [261], and instead of observing lower peak intensities, peak *inversions* indicate the presence of ligand binding [209].

For STD NMR to work properly, the ligand concentration must be in large excess (often 100–1000 fold) over the receptor so that effective saturation transfer can take place [260]. This means that for STD NMR, and WaterLOGSY, only small amounts (µg) of protein are required to get results [261,262,263]. This is advantageous for researchers, as they can perform STD NMR on a protein of interest, and preserve the rest of the unused sample for future/other experiments. Also, the same sample can be used for multiple NMR measurements. STD NMR facilitates the differentiation of binding ligands from non-binding ligands because the change in signal (as determined by the difference spectrum) is easy to measure and observe, as shown in Figure 9. WaterLOGSY has been extended to study ligand interactions with DNA and RNA [261].

There are additional NOE-type experiments (trNOE, INPHARMA, SALMON, etc.) used for drug design, and specific details regarding individual methods are found in the scientific literature [264].

With the pressing search for new antiviral drugs, any techniques for identifying and characterizing novel leads has become increasingly important. Benie et al. [265] described the use of saturation transfer difference (STD) NMR spectroscopy [262,266,267,268,269,270,271] to identify and characterize the binding of an antiviral compound to native human rhinovirus serotype 2 (HRV2). The experiments demonstrated that it is possible to subject targets of the size and complexity of whole viruses (for a model of an HRV2 particle cut open, cf. the table of contents) to STD NMR experiments. The principles of STD NMR have been known for many years [267,268] but it was only recently that the potential of this technique for screening libraries for compounds with binding activity toward protein receptors has been realized [262,266]. The technique also permitted the analysis of epitopes of ligands bound to receptor proteins. Previous NMR studies of virus-ligand interactions used chemical shift titrations, which required very large quantities of the virus. This approach was unworkable when studying pathogenic viruses. Benie et al. [265] demonstrated that solution state STD methodology not only reduces the amount of virus required by approximately 2 orders of magnitude, but also allows for the identification and characterization of virus-ligand interactions with atomic resolution [272].

The very large size of viruses makes them particularly attractive for studies by STD NMR, as they inherently yield large line widths allowing for easy irradiation of the virus without affecting the ligand protons. Furthermore, because of the larger correlation time of a virus in comparison to an average-sized protein, spin diffusion, and thus saturation transfer, is very efficient. The large line width has additional benefits not just for STD-based NMR methods but also for transfer NOESY spectra, as protons from the virus capsid are invisible in the NMR spectra (for an example of a transfer NOESY spectrum, see [265]). Moreover, competitive STD titration experiments can be used to determine the K_d_ value of a ligand [271]. Analysis of the STD spectra using the group epitope mapping method [271] allows for the determination of the binding epitope. STD NMR methods can considerably speed up the determination of the binding epitope for potential antiviral lead compounds.

Simple STD NMR experiments provide substantial information on the binding of ligands to native viruses and require very small amounts of the virus with measurement times in the range of tens of minutes. This allows for a high throughput of ligand samples without significant consumption of viral material because it remains unaffected by the experiments and is easily separated from the low molecular weight ligands by ultra-filtration subsequently. In addition to the detection of binding, a complete mapping of the ligand-binding epitope can be achieved [265].

Noroviruses (NV) are non-enveloped, single-stranded, positive-sense RNA viruses that are the major cause of epidemic outbreaks of gastroenteritis worldwide [273,274,275]. The viral coat consists of a single protein, VP1, which assembles into a capsid with overall icosahedral symmetry [276,277,278]. Attachment of human noroviruses to histo-blood group antigens (HBGAs) is thought to be critical for the infection process [279]. The protruding domains of the VP1 proteins, called P-domains, harbor highly conserved binding sites for HBGAs. STD NMR-based epitope mapping was used [262,271] to identify structural features of different core types critical for the binding of synthetic A- and B-tetrasaccharides [280] to virus-like particles (VLPs) of a highly homologous GII.4 strain (Ast6139). STD NMR experiments provide a robust and straightforward technique for obtaining ligand binding epitopes at atomic resolution. Comparing binding epitopes of related ligands then delivers critical information about structural requirements for ligand recognition. Conversely, comparison of binding epitopes of a given ligand binding to wild type, and to mutant proteins reveals the importance of individual amino acids for binding. STD NMR experiments with L-Fuc and B-trisaccharide in the presence of wild type and mutant VLPs yield virtually identical binding epitopes and suggest that these two mutations do not significantly alter HBGA recognition. The STD NMR approach to characterize binding of HBGA ligands to noroviruses has employed VLPs as targets and thus taken advantage of the large size of VLPs yielding excellent signal-to-noise ratios of the corresponding STD NMR spectra, as demonstrated previously [281].

#### 3.1.4. Transferred NOE (tr-NOE) in Ligand Based Screening

The application of the transferred NOE (Tr-NOE) effect was first demonstrated by Bothner-By [282]. The Tr-NOE is the nuclear Overhauser effect between ligand spins, which are in chemical exchange between the bound and unbound form with the protein or receptor. Ligands, which are a mixture of target molecules, are small in size (below 500–1000 Da). Since they are usually low molecular weight molecules, they exhibit much shorter correlation times when compared to the receptor and have slow NOE build-ups with no spin diffusion. This is the reason they show small positive NOEs in the free form. When binding to a protein receptor, the situation changes, where the ligand acquires large correlation times in the bound state with rapid NOE build-up. Then they show spin diffusion and a strong negative NOE, which is termed the transferred NOE. Signals arising from the protein are usually not observed for large proteins as they are generally kept low in concentration, with ligands in a high excess concentration. In addition, most of the time protein signals are suppressed by their very short T_2_ period. It is worthwhile to mention that ligands that are in fast exchange between the bound and the free form (dissociation constants ranging from μM to mM) get enough bound time to transfer the negative NOE from the protein complex to the population of the free molecules, yet usually retain the chemical shift of the free molecule along with the relaxation characteristics. In order to observe tr-NOEs, the following condition have to be fulfilled:

(3) $$\left|N_{b}\partial_{b}\left|\gg \right|N_{f}\partial_{f}\right|$$

where *N* and ∂ represent the number of molecules and the cross-relaxation rate, respectively. The subscript b and f represent the bound and free form, respectively. Therefore, to observe the tr-NOEs, a high excess concentration of ligands over protein is maintained. On the other hand, if the ligand concentration is kept too high, the excess free ligand in solution will exhibit positive NOE, which can result in a significant reduction of the tr-NOESY enhancements due to negative NOE developed by the very small concentration of bound ligand. Hence, the preparation of the sample becomes tricky and an optimum ratio between 10–30 to 1 is maintained while considering the dissociation constant values. The binding of a ligand to a receptor protein can easily be identified by observing the sign and size of the NOEs. There are some distinct experimental features for the discrimination between tr-NOEs from the bound state and NOEs of the ligand in free states like the build-up rate. For tr-NOEs, this is in the range of 50 to 100 ms, whereas for small ligands it is much longer. There have been various instances of experimental implementations to quickly determine the binding activity of ligand libraries. One example was to find the ligand molecule among a library of 10 similar structure polysaccharides that is bioactive in binding with recombinant E-selectin [283]. This is a protein present in an IgG chimera with a molecular weight of about 220 kDa. In this case, two 2D NOESY spectra were recorded. The NOESY spectra for the ligand library was measured at several temperatures and it was found that most of the 10 compounds exhibited the weak positive NOEs at 310 K, which was then chosen to differentiate between trNOEs showing large negative values. The trNOESY spectra of the ligand library in the presence of protein was recorded at different ratios, such as 5:1, 8:1, 12:1, 15:1, and 20:1, at 310 K. In all the ratios, trNOEs were observed; however, the ratio of 15:1 represented the best-case scenario.

#### 3.1.5. The INPHARMA Method for Pharmacophore Mapping

**Figure 10:**
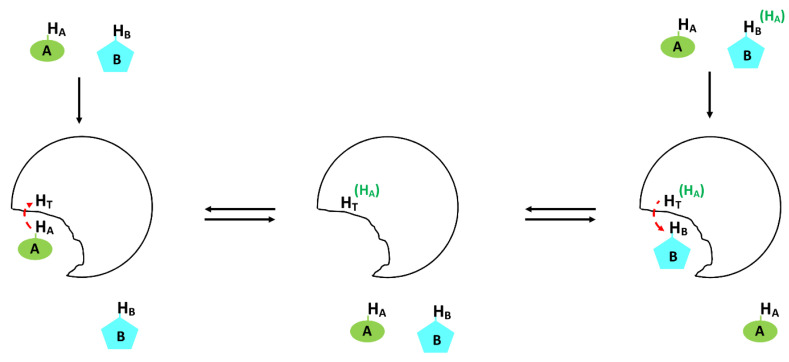
Schematic representation of the principle of the *INPHARMA.* The inter-ligand NOEs observed between two competitive ligands A (in green) and B (in blue), which bind consecutively to the same target receptor T. The figure is adapted from [284].

The INPHARMA method (see Figure 10) was designed to determine the relative orientation between two competitive ligands in the receptor-binding pocket through the observation of inter-ligand NOE between the two ligands. It is a tr-NOE in nature as it is mediated by the bound conformation of the competing ligands and in exchange with the receptor protein. The first example was competitive binding and observation of inter-ligand NOE between baccatin III and epothilone A in the presence of tubulin, which acts as a receptor [284]. Since the observation is on the ligand site, it provides unique advantages. The detailed conformation of a ligand-protein complex can be addressed by conventional NMR. However, it is time-consuming and demands full solving of the structure and there is also a size limitation. From that aspect, ligand-based methods are more useful. The only limiting fact is that it should fulfill all the conditions of tr-NOE explained previously in terms of dissociation constant (*K*_d_), fast exchange regime, and proper ligand to protein ratio. Then, information on the ligand structure can be derived from tr-NOE build up as a function of mixing time. This can be readily explained using the originally proposed schematics [284]. The NOESY spectrum of a mixture of the two ligands A and B in the presence of the common receptor (T) is recorded. Under the situation that each of A and B exhibit competitive binding in a fast exchange regime with the receptor T, intermolecular tr-NOE peaks between the two ligands A and B can then be observed in the NOESY spectrum due to extensive spin diffusion. During the NOESY mixing time, the first proton of ligand A (H_A_) binds to receptor T, which results in transfers of magnetization from H_A_ to H_T_. Subsequently, the complex AT dissociates as they fulfill the dissociation constant range, which creates the opportunity for ligand B to bind to the receptor T at the same binding site. This results in the transfer of the magnetization of H_T_, which had been originally coming from H_A_, to H_B_. As a result, an inter-molecular correlation H_A_–H_B_ can be seen, and this inter-molecular NOE will be a function of mixing time as described above. The detailed analysis of such intermolecular NOE peaks helps in assessing the relative orientation of each ligand in the binding pocket.

#### 3.1.6. Diffusion Based Spectroscopy in Drug Design

**Figure 11:**
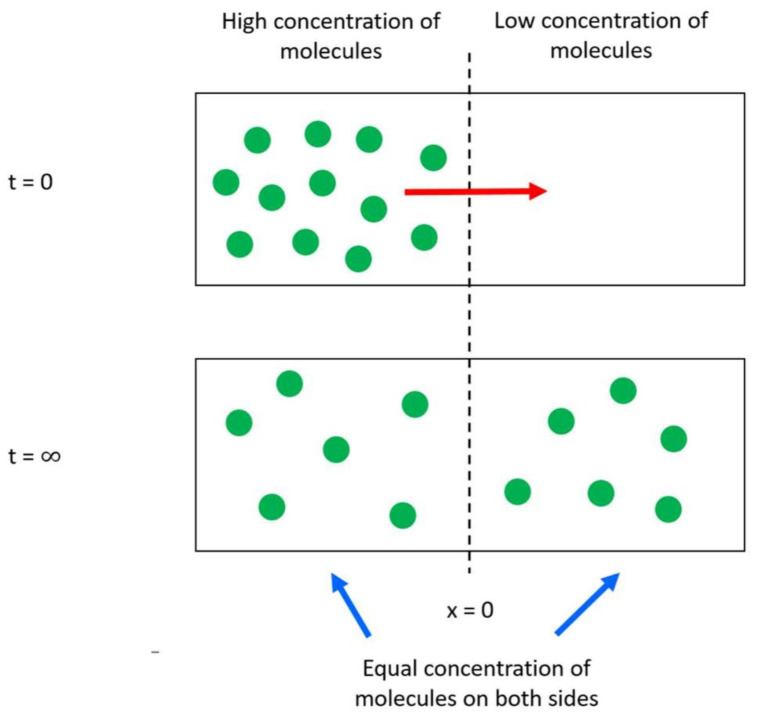
Visual representation of how molecules diffuse in solution from a high concentration to a low concentration across an arbitrarily defined point (x = 0) after some period of time (t = ∞) [289]. The red arrow indicates the direction of translational motion as the molecules (green) move from an area of higher concentration (left) to an area of lower concentration (right) [287].

Diffusion is the random, translational motion of molecules in solution as a consequence of their thermal energy [285]. This type of motion is often referred to as “Brownian motion”, a motion that describes molecular movement induced by random collisions between the molecules [286]. In the presence of a concentration gradient, molecules will naturally move from places of higher concentration to places of lower concentration [287] after a period of time, t, as shown in Figure 11. Fick’s Law can be used to model this type of movement [288]. The distribution of the diffusing molecules is accurately represented by a Gaussian curve, a normal distribution centered at a single point, which gradually “flattens” as t approaches infinity [213]. The extent to which a molecule diffuses is directly related to its shape, size, and mass [285]. In homogeneous isotropic solutions, the root mean square distance (z_rms_) traveled by a molecule is given by following equation [289,290]:

$$z_{rms}={\left(2Dt\right)}^{\frac{1}{2}}\;$$

where *D* is the diffusion coefficient of the molecule, and *t* is the diffusion time. Making the assumption that the molecules are solid rigid spheres, the value of D can be calculated according to the famous Einstein-Stokes equation (Equation (2)):

(4) $$D=\frac{k_{b}T}{6\pi \eta r_{s}}\;$$

where *k_b_* is the Boltzmann’s constant (1.3807 × 10^−23^ J/K), *T* is the absolute temperature, *η* is the solution viscosity, and *r_s_* is the hydrodynamic radius of the molecule [290]. Equation (1) and Equation (2), however, are not universally applicable; they only apply to molecules that are freely diffusing in isotropic, homogeneous solutions, and importantly that can be accurately described as hard, rigid spheres [285]. Different molecular geometries and additional modes of diffusion (i.e., restricted and anisotropic) require more advanced mathematics and theory [291,292], but the essential concepts of diffusion remain the same.

**Figure 12:**
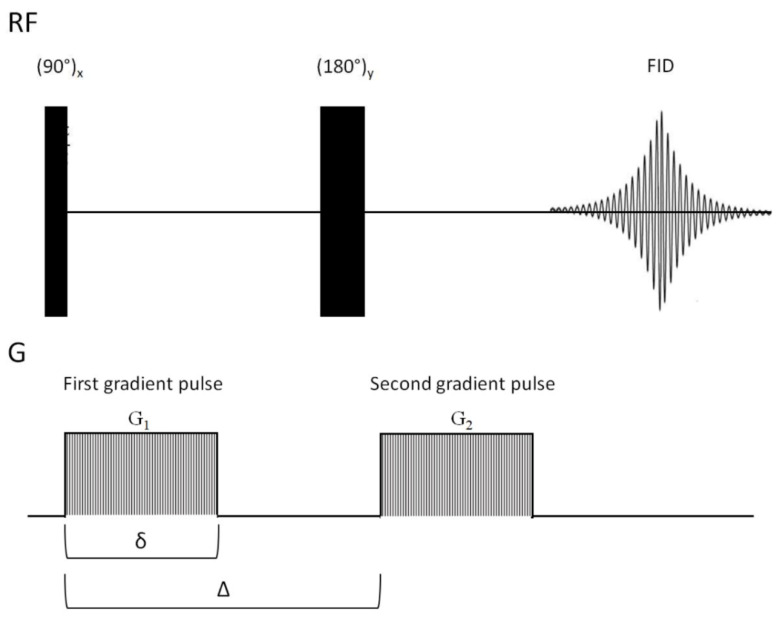
Spin echo (SE) sequence, as discovered by Stejskal et al. [293]. G_1_ is the first gradient pulse applied after the first 90° pulse, and G_2_ is the second gradient pulse applied after the first 180° pulse. δ and ∆ are the gradient length, and diffusion time, respectively.

The earliest pulse sequence used to measure diffusion in NMR spectroscopy is the gradient spin echo sequence (SE), developed by Stejskal et al. [293]. The SE pulse sequence is shown in Figure 12. The SE pulse sequence uses a gradient (G) of the externally applied magnetic field, (pulsed field gradient), the first after the 90° pulse, and the other after the 180° refocusing pulse. The first gradient pulse (G_1_) labels or gradient-encodes the NMR-active nuclei based on their physical position in the sample tube. If the molecules diffuse during the time period they are not in the correct position to experience the second gradient which re-focuses the spins. This is detected via NMR as a signal intensity decrease. After a diffusion time (∆), the second gradient pulse is applied to decode the spatial labeling of NMR-active nuclei, obtaining a well-defined spectra of diffusing molecules in solution [294]. Additional NMR sequences are available for diffusion experiments [295], and are detailed in more comprehensive reviews dealing with the subject [296,297].

The signal intensity of the diffusing molecules depends on three factors, as described by Equation (3) [294]:

(5) $$I=I_{0}e^{-D\gamma^{2}g^{2}\delta^{2}}\;$$

where *I* is the observed intensity, *I*_0_ the reference intensity (unattenuated signal intensity), *D* is, of course, the diffusion coefficient referred to earlier, *γ* is the gyromagnetic ratio of the observed nucleus, *g* is the strength of the gradient, *δ* the length of the gradient, and ∆ the diffusion time [294]. From Equation (3), it is easy to see that the signal intensity decreases exponentially with time, so it is vital to optimize the values of *g*, *δ*, and ∆ for diffusion NMR measurements [294].

The drug design approach based on diffusion NMR is basically a screening technique used to differentiate the binding ligands (drug) from non-binding components [264]. Ligands able to bind should have significantly different diffusion coefficients (D) compared to non-binding ligands [297], i.e., the diffusion coefficients of binding ligands will be smaller than those of non-binding ligands [264]. Thus, diffusion-based NMR is a way of effectively “filtering” and identifying which ligands are binding [264].

Diffusion-based NMR spectroscopy has advantages in ligand based screening applied to drug discovery. For example, Diffusion Ordered Spectroscopy (DOSY) does not require prior separation/purification of the ligand/target solution [298]. Diffusion based NMR allows simultaneous determination of diffusion coefficients in multicomponent systems containing large molecules (i.e., proteins) and possible binding partners (i.e., small drug compounds) [285], and no special labeling or contrasting agents are required, though their use is not exclusively inhibited (for an example of the use of labeled compounds in diffusion NMR spectroscopy, see [299]). A problem occurs when there is significant chemical shift overlap between the binding molecule signals and the target. This situation makes it hard to distinguish the NMR signals [300], and the calculations typically assign an intermediate value to the diffusion rate (i.e., one gets a smear). Multidimensional diffusion NMR pulse sequences are available [301], which may help resolve spectral overlap in 1D experiments [300]. Another issue is that molecules in chemical databases may have generally low solubility [302,303]. Low solubility decreases the overall signal intensity and therefore makes accurately measuring diffusion experiments far more difficult [304].

There are many examples demonstrating the successful application of diffusion NMR in examining drugs of pharmaceutical interest [305], and ligand-target interactions [167]. Hajduk et al. [167] exploited the changes in diffusion rates to detect ligands that bind to the FK506 binding protein and the catalytic domain of stromelysin. Nishimura et al. [306] utilized DOSY, in combination with NOESY to determine the orientation of two guest molecules, *p*-ethoxyiodobenzene and 2-iodo-6-methoxynaphthalene, within a host composed of a tetrakis(4-hydroxyphenyl)-cavitand and a tetra(4-pyridyl)-cavitand.

Furthermore, Matthias et al. [307] used ^1^H molecular diffusion and ^19^F spin diffusion to probe the drug loading properties of the Rf-PEG hydrogel for 5-fluorouracil (FU) and 1,3-dimethyl-5-fluorouracil (DMFU), two anticancer drugs.

DOSY can be combined with Saturation Transfer Difference (STD, discussed earlier in this review) to yield new insights about ligand-target interactions. Kramer et al. [308] combined STD with DOSY to analyze a mixture composed of wheat germ agglutinin and two derivatives of N-acetyl glucosamine (ligands). Using this new technique they were able to obtain high quality spectra of the components in the mixture. Tanoli et al. [309] also combined STD and DOSY to explore the interactions of smaller molecules with bovine serum albumin.

These are just a few examples to show that diffusion NMR spectroscopy has played, and will continue to play, a prominent role in drug design.

### 3.2. NMR and In Silico Screening-Two Complementary Approaches

In silico (virtual) screening is now a standard technique in drug design and discovery [310] that has been in use since at least 1991 [311], though the exact origin of the phrase “in silico” is not clear [312]. The nearly ubiquitous use of virtual screening is due to its efficiency in searching massive chemical databases in order to generate lead molecules [313] that inhibit protein-protein interactions [314], and its ability to help identity ligand (drug) binding sites on the target of interest [310] to lend insight to the mechanisms of action for lead compounds [315,316]. Virtual screening is often accompanied by in vitro or in vivo techniques for pharmacology drug research [312], to increase drug throughput, helping to reduce the time and cost of developing novel drug candidates [317]. Virtual screening has also been used to identify candidates for anti-viral drugs [318] and anticancer drugs [319]. Several chemical databases are available both for public and academic use [320]. Virtual screening is properly identified as a high-throughput screening (HTS) technique [321], though using its full capacity as an HTS technique is not required for most purposes.

Virtual screening requires a minimum of two inputs, (1) a three-dimensional model of the ligand (drug), and (2) a three-dimensional model of the receptor (protein) [322], the latter generated from the atomic studies of proteins via X-ray crystallography or NMR spectroscopy [323]. Virtual screening is not a truly “stand-alone” technique and has often been combined with additional biophysical techniques besides NMR spectroscopy and/or X-ray crystallography [324], such as differential scanning fluorimetry [325], fluorescence polarization, and surface plasmon resonance [324]. In this section, we briefly introduce how virtual screening has been combined with NMR spectroscopy, and how they are complementary approaches to each other in drug design. The complete details of how virtual screening works, and how it applies to drug design outside of its combination with NMR is well documented in additional reviews [310,322,326,327,328,329,330].

A prime example of the complementarity between NMR screening and virtual docking is found in the work of Chen et al. [331], in which the authors sought to target the A_2A_ adenosine receptor (A_2A_AR) protein, a drug target for the treatment of Parkinson’s disease [332]. They used virtual screening and an NMR-based screening method against the same 500 molecules in a fragment library so they could compare the results of both methods. The virtual screen successfully predicted (based on calculated binding affinities) four out of the five orthosteric ligands discovered by NMR that were within the top 5% of the fragment library, showing that the two separate methods can give similar and reliable results. Later on, Chen et al. discovered that virtual screening picked up three additional fragments that remained undetected by the NMR-based method, and were, in fact, A_2A_AR ligands; this shows that though neither method is flawless, they are still perfectly complementary approaches for drug design [322,331].

In another scientific work that integrated NMR with virtual screening, Di Lello et al. [333] found small molecular inhibitors of the enzyme ubiquitin specific protease 7 (USP7), a key regulator of the tumor suppressor protein, p53 [334]. A fragment screen by NMR revealed a series of small molecules that bind in the active site of USP7 near the catalytic cysteine (amino acid 223). A ligand-based virtual screen utilizing the fastROCS program identified ~30 hit molecules, several of which were further characterized by ^1^H-^15^N TROSY chemical shift perturbation and line broadening to probe the binding site of the active hits. Di Lello. also tested the active compounds against EOL-1 cells to verify the hits as identified by virtual screening and further characterized by NMR, showing that the active compounds do indeed inhibit USP7 activity. Through additional study of the active molecules and further optimization of their structures, they eventually discovered a series of ligands that bind in the “palm” region of the catalytic domain of USP7, inhibiting its catalytic activity [333]. This study clearly demonstrates that NMR screening-based techniques can be combined with virtual screening to find viable drugs for targets of interest.

Additional examples of the successful integration of NMR and virtual screening as applied to protein targets are also found in the literature, further demonstrating the practicality and complementarity of virtual screening and NMR [329,335,336,337]. For example, Li et al. [338] used virtual screening filtered by NMR to identify and characterize non-metal chelating metallo-β-lactamase (MBL) inhibitors, and in particular, Verona integron-encoded MBL (VIM)-2, when previously there were no clinically significant inhibitors of MBL, since MBL enzymes hydrolyse many, if not all, β-lactam antibacterials compounds specifically designed to inhibit their activity [339]. Furthermore, Shan et al. [340] and Bertini et al. [337] both used virtual screening and NMR, in their respective studies. Through the combined use of NMR and virtual screening, Shan et al. was able to identify, design, and synthesize novel PDZ domain inhibitors, which are proteins implicated in tumorigenesis [340]. Bertini et al. was able to combine NMR to study the interaction of ligands with metalloproteinases, using known inhibitors of metalloproteinases as a starting point [337]. While HSQC NOESY NMR data provided structural and spatial constraints for the proposed 3D models, virtual screening was used to refine the models, and to probe the ligand-protein interaction. In each case (i.e., ligand-protein interaction), Bertini et al. was able to obtain a well-defined ligand conformation in the protein binding site, thus offering a viable alternative to other approaches described in the literature [337]. Clearly, combining virtual screening with NMR-based methods is advantageous in studying how ligands (drugs) bind and interact with targets (proteins) of interest.

### 3.3. Paramagnetic Resonance in Drug Discovery

Paramagnetic NMR (PNMR) can also play a prominent role in drug discovery [341], as PNMR can provide key structural information in situations where crystal structures cannot due to the weak binding of ligands [341]. PNMR can be used to quantify the binding between ligands and large biomolecules such as proteins, DNA, and RNA [342].

PNMR depends on the presence of a group (called the paramagnetic center) with an unpaired electron [343], and since many naturally occurring biomolecules and organic compounds lack a paramagnetic center, one such as caged lanthanide (CLaNP) [344], must be introduced artificially [341]. Once the paramagnetic center (often a metal ion) is present, paramagnetic effects can be used to measure the distance and the relative orientation (i.e., angle) between molecules [345]. This information is crucial when it comes to determining how ligands and substrates bind. Thus, PNMR is quite a useful technique for drug discovery when a paramagnetic center is present. The most relevant consequence of PNMR for drug discovery is paramagnetic relaxation enhancement (PRE), although there are a number of studies demonstrating the use of pseudocontact shift (PCS) effect in drug discovery research [341].

Paramagnetic relaxation enhancement (PRE) is proportional to the inverse sixth power of the distance between the paramagnetic center and the nucleus of interest (i.e., ^1^H), although it does not reveal anything about relative orientation [341]. PRE can give quantitative information in the range of 10–25 Angstroms [346]. Several researchers have taken advantage of this outstanding property to study the structural and dynamic properties of complex biomolecular machineries in their native environment [347].

For example, Iwahara et al. (2003) demonstrated that a protein’s binding polarity to DNA can be determined by PRE, using EDTA-derivatized deoxythymidine (dT-EDTA) with a chelated metal ion (such as Cu^2+^ or Mn^2+^) as a probe. dT-EDTA with a chelated metal ion is a convenient choice, as it can be inserted into any position of a synthesized oligonucleotide. With data derived from the PRE effect, one can easily determine the polarity of the protein (or drug) binding to DNA [348]. Several researchers have investigated DNA as a drug target [349], and the study of Iwahara et al. clearly demonstrates, and even indicates, that PRE can potentially be used to study the interactions between a drug and DNA [348], provided that a paramagnetic center such as dT-EDTA or a metal ion is present.

Brasuń et al. [350] also used PRE derived distances between a paramagnetic center and a nucleus of interest. They replaced the Cys-S-S-Cys bridge found in oxytocin and vasopressin with the His-Cu^2+^-His motif to investigate if doing so would alter the stability of oxytocin and vasopressin. They determined the distances between the Cu^2+^ ion and 1H nuclei (possible because of PRE), and used these values to generate three-dimensional models of the His-Cu^2+^-His motifs in both oxytocin and vasopressin. In doing so, they indicated that such an approach using PRE can help in designing new biologically active compounds [350], and hence in drug discovery research, as many drug discovery studies require a reliable models for the successful generations of hit-lead molecules, especially in the case of in silico docking [351]. This study again proves the usefulness of PRE, and therefore, PNMR, in drug discovery research.

In two additional studies, Huang et al. [352,353] used PRE in their individual studies of protein binding and protein dynamics, respectively. In the Huang et al. case [352], these authors used PRE to establish a model of the binding between the G-actin protein, and thymosin β4, an actin- binding protein. Using PRE determined constraints (distances) and ^1^H-^15^N HSQC, they were able to establish a well-converging docking structure of the G-actin/thymonsin β4 complex [352]. On the other hand Huang et al. [353] did not measure protein binding, but studied the conformational changes and dynamics of select large membrane proteins utilizing ^19^F-NMR spectroscopy, and Ni^2+^ as the paramagnetic center. Through a series of extensive experiments, they showed that conformational exchange rates of membrane proteins can be determined from measurements of the metal-enhanced longitudinal relaxation (i.e., PRE) of the ^19^F nuclei [353], thus yielding additional information (i.e., protein conformation dynamics) that could be utilized in drug discovery projects targeting proteins (i.e., understanding how the protein changes shape based on its environment can be used to find potential binding sites for drug candidates).

All these examples prove that PNMR is powerful approach in drug discovery research, given that PRE can aid in generating trustworthy models of interacting molecules, and that it can help researchers understand better how the molecules interact in the first place.

### 3.4. Solid State NMR in Drug Discovery

Since the late 1970s solid state NMR (ssNMR) has demonstrated its usefulness in complex biomolecular systems such as collagen or lipid bilayers [354]. However, over the past years ssNMR has gained attention in the field of drug design and is slowly becoming a commonly used technique as its proving to be a powerful tool for structural analysis of membrane proteins and amyloid fibrils [354,355,356].

ssNMR is becoming a more attractive alternative for several different reasons. One of them is the fact that it enables the characterization of a chemical compound in a solid-state form such as in a tablet/pill [356,357,358]. Moreover, ssNMR is not only restricted to analyzing the chemical structure but it can also provide insight into the physical properties of a compound such as polymorphism (different crystalline structures of the same compound), disorder (crystal defects and amorphous solids in the compound) or the presence of cocrystals (multicomponent crystal made of a compound and one or more small organic molecules) [356,357]. ssNMR can also be used to quantify the amount of crystalline against the amount of amorphous material in the sample to establish phase purity (the amount of desired phase separated from other, undesirable phase) [356,357,358].

ssNMR differs from liquid state NMR by the presence of anisotropic interactions. In liquids NMR these effects are averaged to zero as a consequence of rapid molecular tumbling. In solid state however, the molecules are not tumbling rapidly and the residual effects of anisotropic (orientation depended) interactions such as anisotropic chemical shift, magnetic dipolar coupling, and quadrupolar coupling could be observed in the form of broad peaks, with could be much wider than the chemical shift range of the nucleus [355,358,359]. As a results, there has been a constant effort to improve the sensitivity and resolution of solid state NMR spectra, which increased the potential of ssNMR in future applications [360]. One of the methods that works for nuclei with spin value of I = 1/2 is called magic-angle spinning (MAS). It increases the resolution by rapidly rotating the sample around a fixed (or so-called magic) angle of 54.736° [360]. This method can be combined with decoupling, to remove the dipolar couplings between spins. This is done by applying radiofrequency pulses or cross-polarization (CP) transfer of magnetization from abundant and sensitive nuclei such as ^1^H to less sensitive such as ^13^C [328,333]. A broader comparison between ssNMR and liquid state NMR is provided in [361].

As mentioned before, ssNMR can provide information about membranes and membrane proteins. For this reason, ssNMR can be used to detect interactions of ligands with receptors embedded to the membrane which enables the mapping of binding site of a receptor by utilizing CP-MAS (cross-polarization magic-angle spinning) NMR and site specific mutagenesis [355]. ssNMR can provide the conformation of ligands bound to the receptor which can then be used to optimize future drug in terms of better affinity and efficiency [355]. Since ssNMR is also applicable to amyloid research, it can be used for probing polypeptide structures of amyloid and intermolecular contacts between fibrils. The potential is for the design a drug that will inhibit the process of aggregation of proteins and peptides [355]. Lastly, since ssNMR gains insight into physical properties of a chemical compound it can be used for control of the process of formulation and processing of a drug to help assess the purity of a compound [358].

An example of ssNMR application related to drug design is the work of Callari et al., who monitored the effect of drug loading on the properties of micelles [362]. Polymer micelles are widely used as nano-carries for drug delivery, but so far the effects of drug loading on the morphology of a drug carrier had not been thoroughly investigated [362]. They created a model consisting of a fructose hydrophilic block and a PMAA block (micelle), to which a different amount of platinum complex was anchored. The results from this experiment showed that micelles loaded with a higher amount of platinum complex had reduced cellular uptake, release, and cytotoxicity. The micelles with a lower load (LL) of platinum complex were more effective at targeting cancer cells (of cell lines MDA-MB-231 (breast cancer) and A549 (lung cancer) than the micelles with a higher load (HL) of the platinum complex. This is evidenced by the lower IC_50_ (half maximal inhibitory concentration) values of the LL micelles as compared to the HL micelles. Both of those results could be related to the micellar structure and their potential for interaction between the sugar moieties and the cell wall [362].

Another example of practical application of ssNMR is the work of Lee and colleagues [363] in which they investigated the structure of a designed zinc-binding amyloid fibril that catalyzed ester hydrolysis. Metals ions such as zinc where found to affect the process of protein aggregation which resulted in arise of amyloid like structures. Therefore, understanding the processes of aggregation and the factors related to them is crucial for creation of new drugs for amyloid related diseases [364]. In the experiment Lee et al. used Ac-IHVHLQI-CONH_2_ peptide (referred as HHQ) to form fibrils with varying Zn^2+^:HHQ molar ratios. The results showed that Zn^2+^-bound HHQ fibrils form parallel-in-register form of packing β-strand in each sheet and His residues are coordinated to Zn^2+^ via Nδ1, while half of the His residues are also coordinated to Zn^2+^ via Nε2. Additionally, Zn^2+^ binds in a 1:1 metal ion/peptide ratio. After further analysis using structural bioinformatics, it was concluded that each zinc ion was coordinated by three histidine nitrogens from two adjacent strands. Half of all histidines bridged to Zn^2+^ ions forming a metal–imidazolate chain [363].

### 3.5. NMR Validation in Drug Design

A “hit” is a molecule identified from a screening technique (HTS, FBDD, etc) as having a desirable effect (i.e., decreased cellular growth, high affinity score) on a target [365,366]. However, the question of whether the activity is related to actual binding to the target, or to interference with one of the components of the assay readout mechanism, is uncertain. Thus, a validation step is required. Hit-validation is therefore the process of confirming, or validating, that the molecule(s) identified previously have on target activity and selectivity [367,368]. One of the highest-impacts of NMR on drug discovery is the use as a hit-validation tool. Though the hit-validation or confirmation of drugs is mostly limited to the solution state [369], this aspect of NMR truly is a “gold standard” technique in drug discovery.

NMR by itself is a powerful tool for drug validation as in the case of Sharma et al. (2012) [370] who sought to identify potential drug-like inhibitors against L-Aspartate α-Decarboxylase (ADC) an enzyme responsible for the decarboxylation of L-aspartate in order to generate β-alanine and carbon dioxide [371], in *Mycobacterium tuberculosis*. They began with known inhibitors of ADC, and developed a protocol to measure the enzymatic activity of ADC. Upon addition of ADC to a solution of L-aspartate, L-aspartate gradually disappeared because ADC was converted to L-aspartate to β-alanine; therefore the peak intensity of L-aspartate decreased, and the peak intensity of β-alanine increased in the presence of ADC (no inhibitor drug present). Using this newly developed NMR-based protocol allowed direct measurement of ADC enzymatic activity, and Sharma et al. were able to confirm the enzymatic inhibiting activity of seven previously discovered inhibitors of ADC [370]. This study demonstrated that NMR can be an effective validation tool for known drugs and for new drugs generated by a screening approach.

NMR is also able to remove false positives that emerge from biochemical screens [372]. For example, an aptly named technique called A La Assay to detect Reactive Molecules by Nuclear Magnetic Resonance (ALARM NMR) is able to eliminate false positives from HTS methods [373], and in the presence of a test compound or mixture, measures dithiothreitol (DTT)-dependent ^13^C chemical shift changes of the human La antigen [373]. Dahlin et al. provided an updated protocol of ALARM NMR to aid researchers in the production of the ^13^C-labeled La antigen reporter protein, in testing compounds with the La protein, and in the analysis of obtained NMR spectra. Using ALARM NMR prioritized hits identified from HTS screening [374]. An example of ALARM NMR is found in the work of Dahlin et al., where they used this technique to test molecules that were assumed to be inhibitors of histone acetyltransferase (HAT) inhibitors, and from their studies, actually discovered that 65% (15 out of 23) of the most commonly reported HAT inhibitors were actually faulty. They were actually nonselective interference compounds, not necessarily specific to the inhibition of HAT [375]. Thus, ALARM NMR (and NMR in general) served as a useful validation method, especially for unvalidated hits identified from biochemical screens [372] or other screening techniques.

The last example highlights the need for cross validation, or the combination of two or more techniques to verify identified chemical hits. Of course, NMR is not the sole technique used for drug validation. Most often, NMR drug validation is coupled with additional methods [367] such as surface plasmon resonance (SPR) [376,377] X-ray crystallography [377,378,379], isothermal calorimetry (ITC) [379], UV-Vis and/or fluorescence spectroscopy [380].

The work of Goudreau et al. is an excellent example of combining NMR with another biophysical technique, in this case X-ray crystallography, for drug validation [378]. A series of benzodiazepine inhibitors of Human immunodeficiency virus 1 (HIV-1) was identified using an in vitro capsid assembly assay, and further characterized by ^19^F-NMR. Analysis of the chemical shift perturbation and line broadening effect on the ^19^F-NMR spectra of the benzodiazepine inhibitors revealed the specificity and reversibility of the binding inhibitors. The same set of ^19^F-NMR spectra were used to identify the N-terminal domain of the capsid as the binding site of the benzodiazepine inhibitors. The specific amino acids involved in the binding of the benzodiazepine inhibitors were identified from the chemical shift perturbation of ^1^H,^15^N-TROSY NMR spectra. Later, use of X-ray co-crystallography confirmed binding locations of the benzodiazepine inhibitors and their binding modes, which was useful for further development and optimization of the benzodiazepine inhibitors [378]. The work of Goudreau et al. therefore showed how NMR could be used as a co-validation technique with another biophysical method [378].

NMR can be also coupled with multiple biophysical techniques to validate a molecule’s ability to inhibit protein-protein interactions (PPIs) [367]. An example of the combination of NMR with SPR and X-ray crystallography can be found in the work of Fry et al., where the authors sought to understand how the nutlin molecule inhibits MDM2-p53, a protein-protein interaction that has been an important cancer therapy target for several years [381,382,383]. Fry et al. [377] gradually deconstructed RG7112, the first nutlin molecule to enter clinical trials [384], into 11 fragments so they could study the inhibitory effect of RG7112 on the MDM2-p53 interaction by SPR, NMR, and X-ray crystallography. SPR was used to determine the K_d_ values of the RG7112 fragments and confirmed that RG7112 and some of its fragments do bind to MDM2, inhibiting the MDM2-p53 interaction. ^1^H,^15^N-HSQC NMR chemical shift perturbation was also used to assess and verify binding identified by SPR. Of the six fragments of RG7112 confirmed by ^1^H,^15^N-HSQC NMR as binding to MDM2, SPR showed binding for five of them; thus, the two separate techniques were in good agreement with each other. The fragments of RG7112 that were confirmed to bind by both SPR and ^1^H,^15^N-HSQC NMR were further studied with X-ray crystallography, which can tell precisely where and how the molecules bind to the protein. Using co-crystallization, Fry et al. were able to obtain structures for several of the verified binding fragments in complex with MDM2 and were able to visualize the binding of the fragments to the MDM2 protein [377]. NMR is obviously a powerful drug binding validation tool, but it becomes much more powerful when coupled with additional biophysical techniques, as seen in the work of Fry et al. [377].

Dias et al. [379] took a similar approach as Fry et al. [377] in that they took known inhibitors of a protein-protein interaction, and dissected them into individual fragments to assess a protein’s drug-ability. The interaction studied was that between the proteins von Hippel–Lindau (VHL), and the alpha subunit of hypoxia-inducible factor 1 (HIF-1α). Twelve compounds (known inhibitors and derived fragments) were developed using a crystal structure of HIF-1α peptide bound to the stable multiprotein complex pVHL-elongin C:elongin B (VCB). Each of these compounds was screened using three separate NMR techniques, Saturation Transfer Difference (STD), Carr–Purcell–Meiboom–Gill (CPMG) relaxation experiments, and WaterLOGSY (to assess drug binding and to predict drug binding mode. Each compound that was unambiguously detected (i.e., the molecule was identified as successfully binding by at least two of the three NMR methods of STD, CPMG, and WaterLOGSY) was subjected to further analysis by ITC and X-ray crystallography. ITC was used to determine the dissociation constants of binding molecules, and X-ray crystallography was used to confirm the binding mode predicted by the NMR studies. Generally speaking, the designed fragments had similar ligands efficacies compared to the parent molecules but had much higher dissociation constants (K_d_ values), meaning that the fragments bound less tightly than the original parent molecule [379]. With this example, it is possible to see the strength of using NMR as its own hit-validation tool (i.e., three different NMR techniques were used for screening compounds [379]), and yet, the follow-up of NMR studies with ITC and X-ray crystallography was useful in providing a basis for assessing the drug-ability of a protein-protein interaction [385,386,387,388]. Thus, it is clear to see that NMR is a prominent method of hit-validation in drug discovery research, especially in combination with other biophysical techniques.

### 3.6. Other Methods Used to Determine the Drug-Target Complexes

Substantial progress has been made in the NMR field over the past 5–10 years, and various methods were established to determine the drug-target complexes. Most of them utilize either NOE or chemical shift perturbations (CSP) although in silico models/programs, using NMR-derivate data also exist.

#### 3.6.1. DIRECTION

One of the methods called difference of inversion recovery rate with and without target irradiation (DIRECTION) is used to map pharmacophores and can be an alternative to STD experiments. This method uses the difference between longitudinal relaxation rates of ligand protons with- and with-out irradiation of the protons of the target protein. The DIRECTION approach, however cannot be used for slowly exchanging (strong binding) ligands. The practical approach of this method was demonstrated on the experiment when analyzed the interactions between p38 MAPK (p38 a mitogen-activated protein kinase) and its inhibitor-SB203580 [389,390]. The results from this experiment showed that protons H1, H4, H5, and H6 of SB203580, are in close neighborhood with the protons of p38 MAPK when compared with H2, H3, and methyl protons. It indicates that two aromatic rings (a pyridine ring and fluorophenyl ring) of SB203580 interact tightly with p38 MAPK. The results were later confirmed with proton density map of each ligand’s proton, based on the crystal structure of SB203580–p38 MAPK complex [391]. Moreover, the same authors already created a new and improved protein–ligand docking method by combining the DIRECTION obtained NMR data with docking software. [392].

#### 3.6.2. ILOE

A second method that can be used to map pharmacophores is called inter-ligand nuclear Overhauser effect (ILOE). This 2D NMR experiment detects when two ligands bind simultaneously to adjacent sites on a protein surface although both of the ligands do not have to bind to the same binding pocket (opposite to INPHARMA, see above) [5,393]. A negative ligand−ligand NOE signal will be created when ligands bind in close proximity to each other whereas ligands that do not bind will show no NOEs, or at most very weak positive ones [372,394]. ILOE also enables determination of the ligand orientations with respect to one another [393]. As in the case of INPHARMA, ILOE can be utilized even in the absence of a 3D protein structure and used with large proteins. Additionally, ILOE differs from INPHARMA in mixing times—for ILOE the mixing times are typically in the range of 600–800 ms [345]. Application of ILOE was first shown on glycolate+NAD^+^ in the presence of porcine heart lactatedehydrogenase, and by glucose-6-phosphate+NADPH in the presence of *L. mesenteroides* glucose-6-phosphatedehydrogenase and from that time it has been widely used [393,395,396].

#### 3.6.3. SOS-NMR

A third method called structural information using Overhauser effects and selective labeling (SOS-NMR), relies of STD experiments performed on ligand complexes with different protein samples that have been fully deuterated excluding a specific type of amino acid. In other words, the data obtained by SOS-NMR gives insight into the ligand-binding amino acid composition and when taken into consideration the 3D structure of targeted protein can be used to establish the structure of protein-ligand complex. This approach has been demonstrated using two complexes—FKBP complexed to 2-(3′-pyridyl)-benzimidazole and MurA complexed to uridine diphosphateN-acetylglucosamine (UDP-GlcNAc). The results showed that for FKBP and MurA, only four and three amino acids (FKBP: Ile, Val, Leu, Met; MurA: Trp, Phe, His) were needed to be selectively protonated in perdeuterated samples to establish the ligand-binding site. Additionally, on average only 6 amino acids were required for accurate identification of ligand-binding surface. According to authors SOS-NMR can greatly improve the early stages of the drug discovery process [397]. Moreover, combining SOS-NMR with other methods can even further increase chances for a positive outcome of an experiment [398].

#### 3.6.4. Tert-butyl Labelling

A completely different approach to this topic was taken by Chen et al. [399,400]. Instead of using isotope labeling, Chen’s group decided to use a *tert*-butyl group contained within ligand-1 to obtain structural information about the protein-ligand complex [400]. The *tert*-butyl group formed an intense singlet in 1.0 to 1.5 ppm range thanks to rapid methyl rotation and methyl reorientation within that group. When compared with the protein’s ^1^H-NMR signal, the tert-butyl signal tended to be much narrower and resulted in easy detection without the need for isotopic enrichment even in protein complexes of high molecular mass such as *Bacillus stearothermophilus* DnaB hexamer (320 kDa) [399]. Additionally, the *tert*-butyl group produces intense NOESY cross peaks that can be observed even in the situations where normally cross-peaks of the proteins are barely detectable. This is partially because the signal corresponded to nine protons within *tert*-butyl group. Those aspects enable measurements of pseudo-contact shifts generated by paramagnetic tags attached to the protein. As a result, it allows positioning of the ligand on the protein. An example of this approach, is dengue virus NS2B-NS3 protease from serotype 2 (referred as DENpro) in complexed with ligand containing a *tert*-butyl group. The result of this experiment showed NOEs between the *tert*-butyl group of ligand-1 and residue Val155 from DENpro [400].

#### 3.6.5. SALMON

Solvent accessibility, ligand binding, and mapping of ligand orientation by NMR spectroscopy (SALMON) is another method based on the data obtained via nuclear Overhauser effect. This method utilizes WaterLOGSY [401] to probe for solvent accessibility to the ligand and determine the orientation of the ligand by analyzing signal changes in WaterLOGSY spectra (positive signal from unbound ligand vs. negative for protein-bound ligands). This method was first used to determine the orientation of prodrug called tretazicar ((5-(aziridin-1-yl)-2,4-dinitrobenzamide) known as CB1954 in NQO2 (quinone oxidoreductase 2) binding site. Previous attempts had been made to obtain the orientation of tretazicar bounded to NQO2, however the results obtained from X-ray crystallography were inconclusive as two orientations of tretazicar could be possible. The information obtained via SALMON showed that the side chain of asparagine at position 161 formed a hydrogen bond with 2-nitrogroup of tretazicar, and that the aziridine moiety of tretazicar pointed toward the solvent [401].

#### 3.6.6. LOGSY Titration

Another variant of WaterLOGSY method called LOGSY utilizes the titration slopes as a measure of solvent accessibility. The titration slopes are created by a constant increase of protein concentrations. This method also provides more insight into the process of ligand solvation by checking the influence of protein concentration onto the process. This approach was used on the bromodomain 1 of protein 4 (Brd4-BD1) by mapping epitopes of two ligands interacting with Brd4-BD1 and predicting ligands position. The results showed that the triazolopyridazine moiety of both ligands was implanted into the binding pocket of the Brd4. Additionally, the results from LOGSY titration showed that methyl-group 1 of ligand 1, aromatic proton 8 of ligand 2 and aromatic proton 8 of ligand 1 exhibit strong water NOE. This information enabled researchers to utilize a chemical replacement strategy (substitute bound water molecules by suitable functional groups) for aromatic proton 8 in a series of ligands containing the triazolopyridazine ring. Those protons were replaced with an amino or aminomethyl groups and as a result, the binding affinity of those ligands increased 100-fold. Finally, the results obtained from X-ray crystallography for ligands with such modifications allowed to find the binding mode of the triazolopyridazine ring of ligand 1 (with methyl group pointing internally) and the substituted amino group was found to create hydrogen bond to the side chain of Asn140 of Brd4-BD1 [402].

#### 3.6.7. Nuclear Magnetic Resonance Molecular Replacement (NMR^2^)

The most recent approach called Nuclear Magnetic Resonance Molecular Replacement (NMR^2^) utilizes spatial data obtained through solution-state NMR in order to locate the binding pocket of a complex structure. For that, it uses a receptor model, e.g., a X-ray structure of a homolog, to conduct an analysis and at the same time excluding the need for protein resonance assignment. To conduct an experiment using such an approach requires a few steps. First, either the protein or ligand used in the complex must be uniformly ^13^C and ^15^N labeled. Then, an experiment to assign the ligand is needed such as 2D ^13^C,^1^H-HMQC or ^13^C,^1^H-HMBC. The next step is the evaluation of ligand intra- and ligand–protein intermolecular distances through NOE cross peaks obtained from F_1_-^15^N,^13^C-filtered ^1^H,^1^H-NOESY. Lastly, choosing a proper input structure is required which can be either X-ray or NMR structures in apo form, with another bound ligand, or a homolog to the protein of interest. Then the NMR^2^ program analyzes for all possible partial assignments (such as methyl groups of a protein) and calculates the complex structures for all options [403,404]. This method was already successfully used to resolve complex structures in case of slow and fast exchange ligands [403,404,405,406].

#### 3.6.8. HECSP

In silico methods combined with NMR derived information can also be used to determine accurate drug-target complexes. ^1^H empirical chemical shift perturbation (HECSP) is an empirical model that is based on chemical shift perturbation (CSP) of a protein. CSP represents the change in chemical shifts in a protein due to alteration of its chemical environment (which can happen upon ligand binding). The CSP of a target protein is obtained by a series of 2D HSQC experiments with a set of ligand titrations involving samples that contain ^15^N-labelled protein. The calculation of ^1^H-CSPs inside the protein are based on four contributors: 1) ring current, 2) electric field, 3) hydrogen bonding, and last 4) magnetic anisotropy. To show the value of the HECSP model two CSP examples were used: apo-neocarzinostatin (apoNCS)-naphthoate ester complex, and human intestinal fatty acid binding protein (hIFABP)-ketorolac-ANS complex. The results from the experiment showed that HECSP model can distinguish native ligand from decoys and more clearly define protein-ligand complex structures with NMR derived information [407].

#### 3.6.9. SAMPLEX

Another program that can utilize CSP called Smoothed Automatic Mapping of Protein from Listed Extremes (SAMPLEX) can help to determine the interaction surface of proteins complexes. SAMPLEX takes the chemical shifts of the protein of interests in both the free and bound state and corresponding 3D structure of a protein in the free state. The programs returns a confidence value for each residue to be in a perturbed or unperturbed state (0.05 as being in a perturbed state, −0.05 as remaining in their unperturbed state). This approach was tested on five examples, one of which was Subtilisin BPN’ (serine protease) complexed with its inhibitor–chymotrypsin inhibitor 2. The results showed that residue 2, and residues 56–62 of chymotrypsin inhibitor-2 were perturbed and residue 63 was in an ambiguous state. To compare, the X-ray crystallography data showed residues 50 and 54–61 to be involved in the interaction. For subtilisin BPN’ the program predicted residues 33, 97, 99–109, 126-128, 141, 154–156, 167–171 and 218–219 to perturbed and residues 65, 98 and 220 to be in ambiguous state. That information was also confronted with the X-ray crystallography data which shown residues 99–104, 125–128, 154–157, 167, 218–221 to be perturbed [408].

## 4. In-Cell NMR Approaches

The interactions between targets (proteins) and ligands (small molecules) can be analyzed independently of the biological systems by using ‘cell-based’ NMR drug design approaches. Three basic approaches [409] are as follows: (1) Compound-detected *in-cell* NMR, (2) Target-detected *in-cell* NMR, and (3) Reporter-detected *in-cell* NMR. 

**Figure 13:**
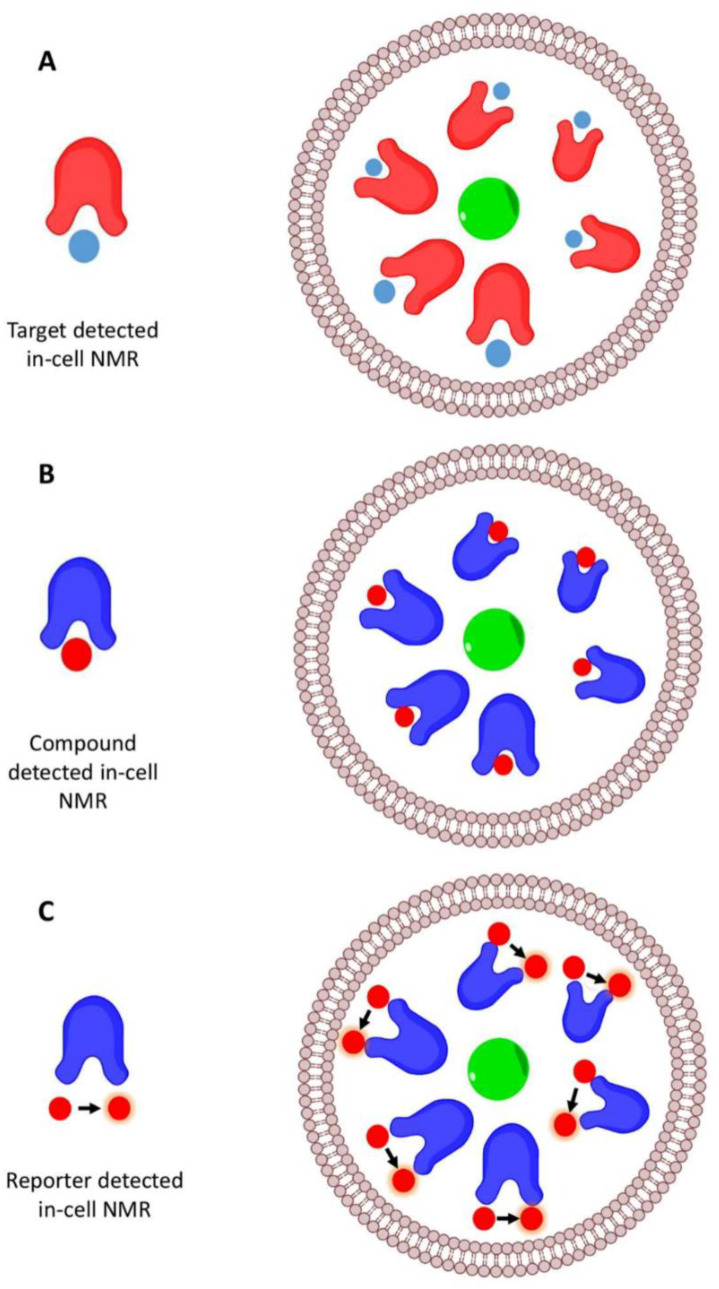
Representation of different in-cell NMR techniques. (**A**) Target-detected in-cell NMR, (**B**) compound-detected in-cell NMR, (**C**) Reporter-detected in-cell NMR. The red color is the isotopically labeled component of the system. Adapted with permission from [409].

These methods, with the exception of compound detected *in-cell* NMR, differ according to the isotopically labeled structure (protein, cell structure, etc.), which enables NMR detection. A cartoon representation of each of these methods is given in Figure 13.

### 4.1. Compound-Detected In-Cell NMR

STD NMR is a technique that lies within the compound-detected *in-cell* NMR method but does not require isotope-labelling of the studied compound. However isotopic labelling of the compound may be used to enhance the quality of the spectra.

### 4.2. Target-Detected In-Cell NMR

In the target-detected in-cell NMR only the target of interest is isotopically labeled (i.e., ^15^N labeled protein). For instance, target proteins can be isotopically labeled during cell growth in isotopically enriched (^13^C, ^15^N, or both ^15^N/^13^C) media [410]. The cell type and the labeling method may vary across experiments. Different cell types, including bacteria [411], oocytes [412], yeast cells [413], mammalian cells [414], HeLa cells [415] and even insect cells [416] have been reported in the literature. The fact that *in-cell* NMR applies to more than one cell type testifies of the versatility and potential application of this technique.

In terms of labeling, ^15^N is one of the most commonly used approaches [417] when the targets of interest are proteins. Recently, ^19^F labeling has been reported as a useful probe for protein-ligand interactions [418]. It was shown that ^19^F can reveal information about the dynamics of protein-ligand interactions [419]. Methyl groups [420] have also been used as probes for proteins and complexes in vivo [420], proving that labeling specific chemical groups instead of the entire biomolecule (i.e., protein) is feasible.

### 4.3. Reporter-Detected In-Cell NMR

In-cell NMR extends beyond proteins, and has been applied successfully to DNA [93,421] and RNA molecules [422,423]. Telomeric repeats have also been studied using target detected *in-cell* NMR [424]. The reporter-detected *in-cell* NMR technique isotopically labels neither the ligand nor the target, but rather a receptor that indirectly measures the effects of ligand-target binding [409].

The “reporter” varies according to the experimental context. For instance, Dose et al. [425] used acetylation- and deacetylation-based assays to monitor the activity of histone deacetylase and acetyl-transferase. Thongwichian et al. [426] used peptide-based reporters to identify active kinases and phosphatases in cellular conditions. Lastly, Doura et al. [427] designed a ^19^F probe that operates in biological conditions in order to study the adherence and dynamics of proteins found in human blood.

### 4.4. “In-Virus” NMR Strategy

In many viruses and phages, scaffolding proteins (SPs) are required to ensure the correct organization of coat proteins (CPs) and other minor capsid proteins into a precursor structure, called a procapsid [428,429]. Although SPs are critical for viral assembly and therefore potential therapeutic targets their structural properties (with only a few exceptions [430,431]) are poorly understood. The size limitation of NMR can be used advantageously as a filter to identify disordered segments even in very large supramolecular protein complexes. In this way, NMR can provide a unique perspective on the dynamic and disordered elements of macromolecules not accessible by other techniques. The procapsid encapsulation experiments described by Whitehead et al. [432] were conceptually analogous to *in-cell* NMR experiments [433,434,435] in which signals from small proteins, or flexible segments of proteins, can be observed when they are incorporated inside living cells, as long as the isotope-labeled proteins of interest do not interact strongly with other large cellular components [433,434,435]. The so called ‘‘in-virus’’ NMR strategy applied by Whitehead et al. [432] could be more generally used to study the dynamic properties of macromolecules encapsulated into virus particles, including cargo molecules encased in viral capsids for nanotechnology applications. Additionally, such studies could assess the level of interaction of cargo molecules with the virus and probe the release properties of cargo NMR [432].

## 5. Final Remarks

As we have attempted to emphasize and demonstrate, NMR has a powerful and unique role in drug design. NMR provides detailed structural information about a molecule along with kinetic information over extended time periods, i.e., not just a snapshot [24,25]. Moreover, NMR is quantitative and highly reproducible, allowing applications in diverse fields such as relaxometry, combinatorial chemistry, fluxomics, and targeted analysis [91]. NMR can be combined with other analytical techniques, such as mass spectrometry “in tandem” analysis of the molecules of interest [436,437,438]. ^1^H 1D-NMR is used particularly in the analysis of metabolites, while the strength and intensity of the recorded signals is directly proportional to the concentration of the sample [44,439,440]. ^1^H 1D-NMR can also be used to follow “real-time” analysis of different molecules [441]. In the 1D ^1^H-NMR experiment, there are no polarization transfer techniques required (the ^1^H atom is already highly sensitive) and covers a spread of interesting nuclei in the molecule(s) being studied [91].

Assuming that the sample can be stored stably for extended periods of time, the non-destructive nature of NMR permits the re-use on the sample for different experiments [50,442]. Aiding in the reproducibility of NMR [91], sample-recycling offers a significant advantage of NMR [443,444].

While always important in drug design, sensitivity and resolution of NMR are two major factors that need special consideration. Since both factors improve with increasing magnetic field strength, we have seen a spike in the demand for ultra-high-field NMR spectrometers. Recently, 28.2 T (i.e., 1.2 GHz for ^1^H) magnets have become commercially available, and with recent advances in magnet technology, such as liquid helium recycling and magnetic field shielding [91], NMR has begun to offer far better resolution and higher sensitivity while reducing the substantial costs of maintaining the instruments compared to past decades. Other steps have been taken to enhance the sensitivity of NMR including: the development of cryoprobes increasing the signal to noise ratio 3 to 4 times, and micro-coil probes that not only increased sensitivity but also reduced the amount of sample required for the measurements [445].

From another perspective, one can further optimize the process of obtaining spectra by using different methods of measurements. One of these, called SOFAST, helps to reduce the delay between scans resulting in lowering acquisition time for 2D experiments such as HMQC utilizing ^1^H,^15^N or ^1^H,^13^C [117,446,447]. The basic principle of this method is to use selective ^1^H pulses that will excite only a small portion of the available nuclei pool, while the unperturbed spins provide a magnetization “heat sink” thus improving the spin-lattice relaxation (T_1_) rate via dipolar interactions [446]. These methods can be highly efficient when studying drug binding and molecular interactions [117].

Another method, called ultrafast 2D NMR, enables obtaining a 2D spectra within a single scan but with the associated cost of reduced sensitivity [117,448]. The principle of this method is to divide the sample into *n* number of fractions, and apply the appropriate incremental aspect to each fraction, while recording them all simultaneously within the one scan [448]. This method has been applied to many metabolomic studies [177,447,449] as well as in the analysis of natural products [450]. The difficulty is that the effective concentration of the sample is lowered by the fractionation level. The more slices the sample is split into, the greater the reduction of combined signal that is obtained.

Lastly, a method termed non-uniform sampling (NUS) may provide an advantage by reducing the total time for measurement while maintaining the same resolution of spectra [117]. NUS effectively skips over parts of the total dataset, collecting only around 20 to 30% of the total. Usually sections containing higher concentrations of signal (over noise) are emphasized in the selection scheme, known as non-linear data acquisition. These methods reconstruct the complete data subset by applying various algorithms such as multidimensional decomposition (MDD), which essentially separates the sets of multidimensional data into one dimensional problems that are much easier to solve given the common process of signal overlapping in multidimensional NMR spectra [117]. Other algorithms such as compressed sensing (CS), Maximum entropy method (Max Ent) and Iterative soft threshold (IST) each have their own advantages but all focus on decreasing the time needed for collection of spectra [117,451]. The cost is in signal to noise, as no gain is ever absolutely free.

**Scheme 2:**
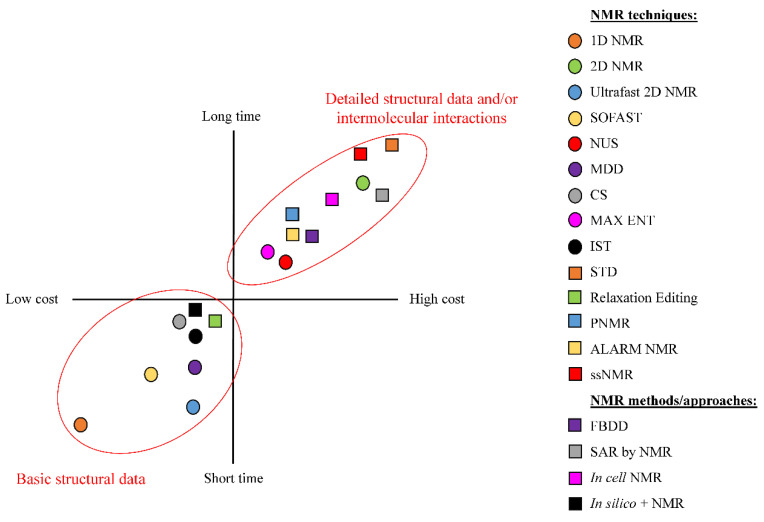
Analysis of the cost and time between different NMR methods used in drug design. Meaning of some of the acronyms are listed below: SOFAST—Band-Selective Optimized Flip Angle Short Transient; NUS—Non-uniform sampling; MDD—Multidimensional Decomposition; CS—Compressed Signaling; MAX ENT—Maximum Entropy; IST—Iterative Soft Threshold; STD—Saturation Transfer Difference; PNMR—Paramagnetic NMR; ALARM—A La Assay to detect Reactive Molecules; ssNMR—Solid State NMR; FBDD—Fragment Based Drug Design; SAR—Structure Activity Relationship.

The inherent advantages of NMR do not eliminate the disadvantages (Table 3), which are namely being limited for some nuclei, and inherent insensitivity for many types of experiments. NMR can provide high quality resolution and sensitivity for some experiments [91,452] but the application can be challenging. When the experiments become multidimensional, there is often a tradeoff between the improved higher resolution and/or the resulting sensitivity and/or the amount of time an experiment takes, i.e., high-quality spectra for multidimensional experiments take much longer than their simple 1D counterparts [117] (Scheme 2). One must always choose between resolution, sensitivity, and the amount time; as you can only ever have two out of the three.

Unfortunately, in the real world, when working with NMR spectroscopy, we are mainly forced to choose between high or low precision data (Scheme 2) with fixed available instrument times. Scheme 2 can be useful, when choosing NMR technique and/or method/approaches in drug studies. It shows the general representation of different NMR techniques and method/approaches in cost-time matrix, bearing in mind that the exact position in the matrix can be influenced by the environmental conditions, pulse sequence and sample preparation (e.g., concentration). Regarding, NMR methods/approaches, the exact position depends on the choice of proper NMR technique. For the purposes of Scheme 2, the 2D techniques were chosen as described in the practical examples. Nevertheless, with relatively short times and at low cost, we can acquire numerous data sets and therefore the low precision can be partially compensated for by statistical analysis.

Fortunately, significant efforts have been undertaken to reduce the amount of time it takes to record multidimensional spectra, especially for 2D NMR, and still obtain high quality spectra (Table 3). Novel pulse sequences have been developed to decouple nuclei in Pure-shift NMR [117]. Dynamic Nuclear Polarization (DNP) can induce hyper-polarization in atoms (^13^C, ^15^N) with an inherently low sensitivity [453,454]. Parahydrogen-Induced Polarization (PHIP) and Signal Amplification by Reversible Exchange (SABRE) are other polarization techniques used to increase the sensitivity of inherently insensitive nuclei [455].

## 6. Conclusions

NMR has become a “gold standard” method in drug design due to its speed, simplicity, and reproducibility. The standardized ppm scale allows one to compare all NMR results and gather them in databases for the common use of researchers. Although sample labeling was limiting in the beginning, it has now become a strength of NMR that permits the observation of big molecules and/or biomolecular processes “through the door lock”. NMR is the only analytical technique that permits qualitative and quantitative analysis without previous sample purification or separation. High precision NMR data still requires long experiment times and has elevated costs; however, these will no doubt be alleviated in the future.

## Figures and Tables

**Table 1:** Recent pharmacological studies complemented with NMR approaches for evaluating the efficacy/safety of new or existing drugs.

Tested Substance	Evaluated Effect	NMR Experiments Used in the Study	Ref.
**Isoniazid (INH)**	INH induces oxidative stress, disturbs energy metabolism, and causes disorders in neurotransmission and neuromodulation processes in Sprague Dawley rats.	^1^H-NMR CPMG ^1^H,^1^H-TOCSY ^1^H,^13^C-HSQC	[178]
**Naproxen**	Naproxen induces a disturbance in energy and choline metabolism, and promotes catabolism of tryptophan in Sprague Dawley rats.	^1^H-NOESYPRESAT-1D	[179]
**Cisplatin (CP)**	Identification of six serum (alanine, betaine, glucose, glutamine, lactate, and leucine) and eight urinary (alanine, acetate, citrate, glucose, glycine, guanidinoacetate, hippurate, and lactate) metabolites that can be used as biomarkers for cisplatin nephrotoxicity.	^1^H-NMR	[180]
**Shell of *Herpetospermum caudigerum* Wall (SHCW)**	A high dosage of SHCW causes the disturbance of energy and amino acid metabolism and induces oxidative stress in Sprague-Dawley rats.	^1^H-NMR CPMG ^1^H,^13^C-HSQC ^1^H,^13^C-TOCSY	[86]
**Ampicillin, Maculatin 1.1**	Both antibiotics cause destabilization of membrane integrity and increase breakdown of nucleic acids in *E. coli*.	^1^H-^31^P CP	[94]
**Emodin**	Emodin can affect the immune response and interrupt energy metabolism (citric acid cycle) along with glutathione synthesis, which can lead to oxidative stress.	^1^H-NMR CPMG ^1^H,^1^H-COSY ^1^H,^13^C-HMBC ^1^H,^13^C-HSQC	[65]
**“RenqingMangjue” pill (RMP)**	RMP can disturb the citric acid cycle in cells, and decreases levels of glutamate, glutamine and BCAAs in the plasma of Wistar rats.	^1^H-NMR CPMG ^1^H,^1^H-COSY ^1^H,^13^C-HSQC ^1^H-^13^C HMBC	[66]
**Ptac2S**	Ptac2S limits cancer cell (Skov-3) proliferation by reducing the efficiency of the citric acid cycle and induces changes in cell membranes in a shorter period of time (6h) compared to cisplatin (24h). Additionally, Ptac2S may inhibit lactate dehydrogenase.	^1^H-NMR CPMG ^1^H,^1^H-COSY ^1^H,^13^C-HSQC ^1^H,^13^C-HMBC	[145]
**Gemcitabine-carboplatin (GC)**	Identification of two biomarkers (formate and acetate) that can predict a positive response in MBC (metastatic breast cancer) patients treated with GC chemotherapy.	^1^H-NMR CPMG ^1^H JRES ^1^H,^1^H-COSY ^1^H,^1^H-TOCSY ^1^H,^13^C-HSQC ^1^H,^13^C-HMBC	[137]
**Doxorubicin (DOX)/dexrazoxane (DEX)**	DOX decreases ATP production and induces oxidative stress in H9C2 cells. DEX counteracts those changes, having a cardioprotective effect on H9C2 cell lines.	^1^H-NOESYPRESAT-1D	[67]
**Curcumin**	Curcumin shows antihyperlipidemic effects on C57BL/6Slac mice by partially restoring metabolic defects induced by a high-fat diet. Affected metabolic pathways include the citric acid cycle, glycolysis and gluconeogenesis, ketogenesis of BCAA, synthesis of ketone bodies and cholesterol, and choline and fatty acid metabolism.	^1^H-NOESYPRESAT-1D ^1^H-^1^H TOCSY ^1^H,^13^C-HSQC	[136]
**Formosanin C (FC)**	Formosanin C shows the ability to inhibit synthesis and methylation of DNA as well as reducing the activity of the citric acid cycle and energy metabolism in the mitochondria of HepG2 cells.	^1^H-NMR	[181]
**Melamine**	Melamine disrupts metabolism of glucose, nitrogen, and protein in the liver of Wistar rat.	^1^H-NMR CPMG	[182]
**Aristolochic acid (AA)**	Aristolochic acid causes renal lesions and a disorder in tubular reabsorption in Wistar rats.	^1^H-NMR	[68]
**Rituximab**	Evaluating response outcome for patients with rheumatoid arthritis, treated with rituximab. Identification of metabolites changes between responders and non-responders such as succinate, taurine, lactate, pyruvate and aspartate.	^1^H-NMR	[183]
**Levetiracetam, Lamotrigine, Topiramate**	No distinction between metabolite profiles of serum from patients treated with levetiracetam, lamotrigine and topiramate. Could not evaluate response of initial treatment of epilepsy.	^1^H-NOESYPRESAT-1D	[184]
**Hexacationic Ruthenium Metallaprism**	Metallaprism mainly affects lipid metabolism in A2780 (human ovarian cancer) and HEK-293 (human embryonic kidney) cells, and increases GSH levels in all cell lines. In A2780cisR (cisplatin resistant A2780) cells, lipid biogenesis and glycosylation are affected by treatment with metallaprism.	HR-MAS: a)1D -^1^H NOESY -^1^H CPMG b)2D -^1^H,^1^H-TOCSY -^1^H J-resolved	[138]
***Centella asiatica* extract**	Extract from *Centella asiatica* promotes glycolysis, boosts the citric acid cycle and decreases gluconeogenesis and lipid metabolism in T2DM Sprague–Dawley rats.	^1^H-NMR CPMG	[185]
**VR24, VR27 (1,3,4-thiadiazoles)**	VR24 and VR27 improve glycerol metabolism, decrease betaine levels, and normalize the altered level of myoinositol in serum of DMH-induced CRC (colorectal cancer) Wistar rats.	^1^H-NMR CPMG	[186]
**Xiaoyaosan**	Xiaoyaosan regulates energy metabolism, can play an important role in the regulation of the nervous system, and might restore the balance in gut microbiota of depressed patients.	^1^H-NMR CPMG ^1^H,^1^H-COSY ^1^H,^13^C-HMQC	[187]
***Sini* decoction (SND)**	SND may restore balance in myocardial energy metabolism, and regulate the citric acid cycle and amino acid metabolism. Identification of 10 biomarkers showing potential efficiency of SND administration in Sprague-Dawley rats.	^1^H-NMR	[188]
***Fu Fang Jin Jing Oral Liquid (FJJOL) with Herba Rhodiol***	FJJOL regulates energy metabolism of brain tissue, can affect the function of neurons abundant in GABA and glycine receptors, and may help to maintain the membrane integrity of the cells in Kunming-strain mice exposed to hypobaric hypoxia.	^1^H-NOESYPRESAT-1D ^1^H,^1^H-gCOSY ^1^H,^1^H-TOCSY	[87]
**Acyclovir, Pyrazinamide, Isoniazid, Sulfamethoxazole**	Evaluating the efficacy (determined by the concentration of a drug able to reach the therapeutic site) of four drugs in cerebrospinal fluid of tuberculous meningitis patients.	^1^H-NOESYPRESAT-1D ^1^H,^1^H-COSY	[189]
**Genipin**	Genipin can recover energy metabolism to normal levels, and regulate methylamine and amino acid metabolisms of diabetic Sprague Dawley rats.	^1^H-NOESY-1D	[190]
**Adriamycin (ADR)**	Identification of seven biomarkers: trimethylamine oxide (TMAO), taurine, trimethylamine (TMA), hippurate, trigonelline, citrate and 2-oxoglutarate that can predict tumor’s (gastric adenocarcinoma) response to ADR treatment in BALB/c-nu/nu mice.	^1^H-NOESYPRESAT-1D	[191]
**Danggui/European Danggui**	Comparison between Danggui and European Danggui showed that Danggui has a different chemical composition and provides a better enriching effect on blood than European Danggui. Identification of 18 metabolites affected by Danggui treatment.	^1^H-NMR CPMG ^1^H-NOESYPRESAT-1D ^1^H,^1^H-COSY ^1^H,^13^C-HSQC	[88]
**Erythromycin**	Erythromycin decreases citric acid cycle activity, enhances fatty acid oxidation, causes dysfunction in amino acid metabolism, and creates oxidative stress in livers of Wistar rats.	^1^H-NMR CPMG ^1^H-NMR BPPLED	[192]
**Fuzi/Gancao**	Fuzi causes a shift in energy metabolism (from aerobic respiration to anaerobic), induces membrane toxicity, and disrupts the balance of gut microbiota of Wistar rats. Administrating Fuzi with Gancao diminishes the toxic effects of Fuzi.	^1^H-NOESY-1D	[193]
**Kijitsu, Tohi, Chimpi,** **Kippi, Seihi**	^1^H-NMR spectra enabled the identification of three compounds (naringin, sucrose, and β-glucose), and ^13^C-NMR enabled the identification of eight compounds (naringin, neohesperidin, ɑ- and β-glucose, sucrose, limonene, narirutin, and synephrine).	^1^H-NMR ^13^C-NMR	[112]

**Table 2:** The names, structures, targets, FBDD optimization strategy used, biophysical techniques used, and status in clinical trials of select drugs derived from the FBDD approach.

Drug (Company)	Target	Original Fragment(s) *	Advanced Molecule and Progress in Clinical Trials	Techniques Used
Vemurafenib (Plexxikon) [224,226]	BRAF-V600E	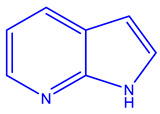	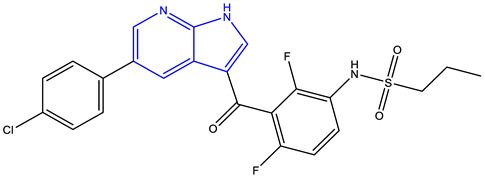 Approved	high-concentration biochemical fragment screening, X-ray crystallography
Venetoclax (AbbVie, Genetech) [227,228,229,230]	BCL-2	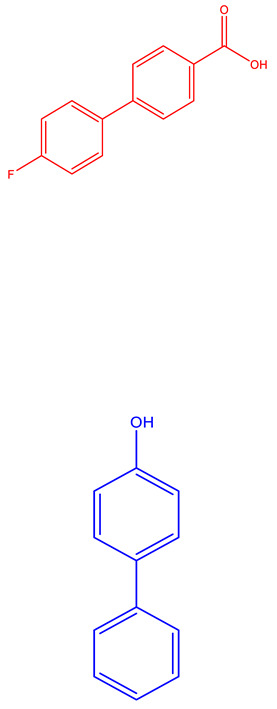	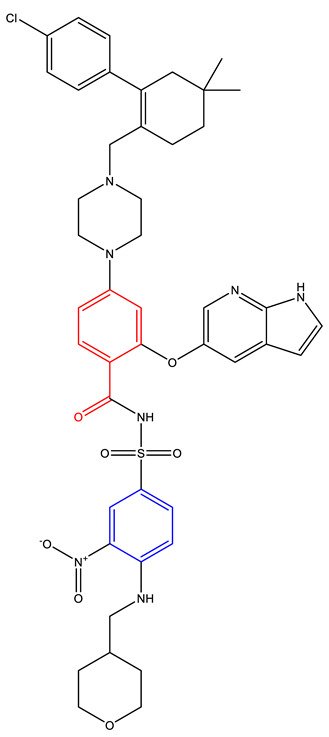 Approved	NMR, X-ray crystallography
Ribociclib (Novartis Europharm Limited) [231]	CDK4 and 6	Information Not Available	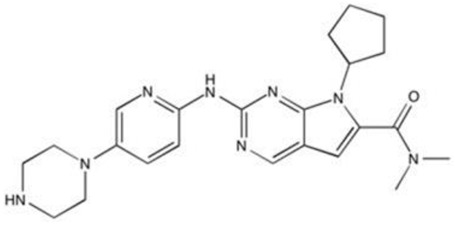 Approved	Information not Available
PLX3397 (Plexxikon) [232,233,234]	FMS, KIT, and FLT3-ITD	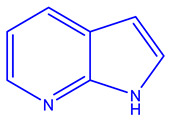	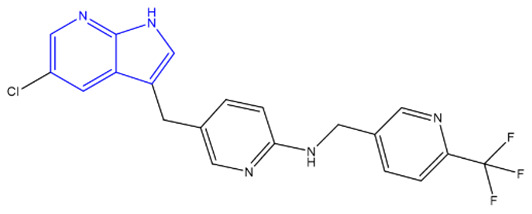 Phase 3	X-ray crystallography, Structure Confirmed by NMR, MS, and HPLC
Verubecestat (Merck) [235,236]	BACE1	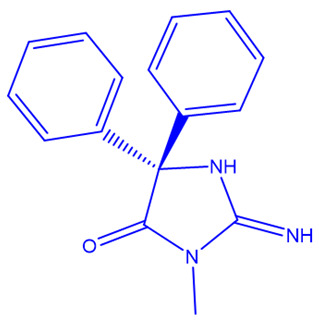	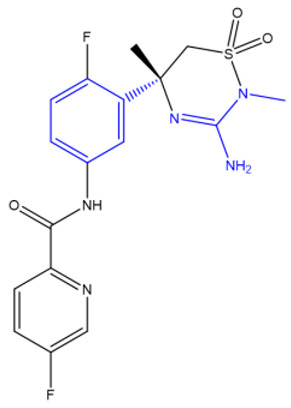 Phase 3	NMR, X-ray crystallography, inhibition of cathepsin D
Onalespib (Astex) [237,238]	HSP90	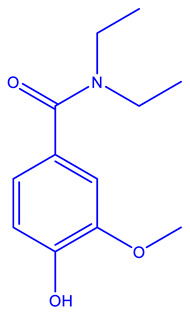	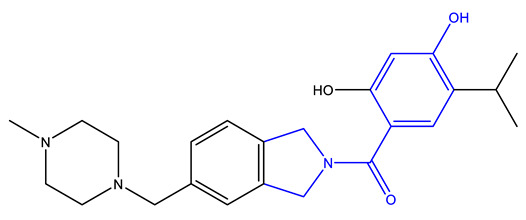 Phase 2	X-ray crystallography, isothermal titration calorimetry, NMR
AZD5099 [239,240]	Topoisomerase II	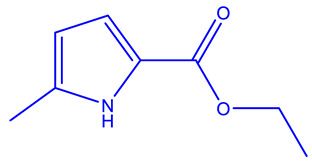	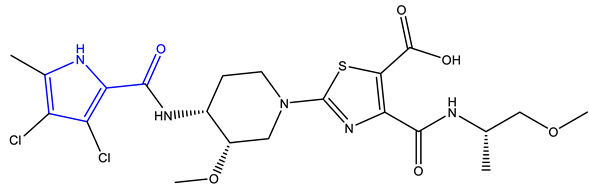 Phase 1	NMR, Surface Plasmon Resonance, isothermal calorimetry, X-ray cystallography
AT7519 [241,242,243,244]	CDK 1, 2, 4, and 5	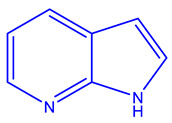	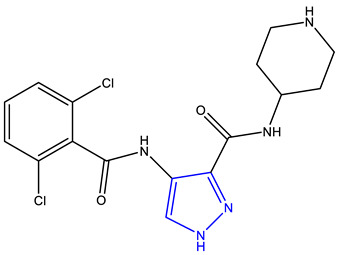 Phase 2	NMR, MS, X-ray crystallography

* Structures for some of the fragments are taken directly from Dan Erlanson’s blog at [245].

**Table 3:** Advantages and limitations of different NMR techniques developed in recent years.

NMR Technique	Advantages	Disadvantages
**1D-NMR**	Ability to identify simple chemical compounds. High quality resolution and sensitivity for many 1D experiments. Less time consuming compared to 2D NMR.	Compared to 2D NMR less details can be obtained for more complex molecules. For nuclei other than ^1^H and ^19^F, relative sensitivity is fairly low—requires extra labeling to obtain better spectra.
**2D NMR**	Ability to identify complex molecules and observe different interactions between the nuclei, e.g., correlations between all spins in one spin system using TOCSY experiment.	Requires long times to obtain a proper spectra (up to days).
**Ultrafast 2D NMR**	Greatly reduces time to obtain 2D spectra.	Reduced sensitivity.
**SOFAST**	Significantly reduces acquisition time of HMQC.	Relatively low resolution
**NUS**	Lowers the time of measurement while keeping the same level of resolution.	Requires use of reconstruction algorithms since missing data points can lead to artifacts in spectra.
**MDD**	Multidimensional data are “broken” to one dimensional, which are easier to analyze. Ability to resolve overlapping resonances.	Data must be (approximately) symmetrical (Lorentzian shape) to obtain good spectra.
**CS**	Good reconstruction of weaker peaks.	Large computational costs, low performance on noisy data.
**MAX ENT**	Significant reduction in acquisition time.	Nominally Lorentzian peak shapes may be distorted, and peak intensities may be altered.
**IST**	Greatly reduced time to obtain NMR spectra.	Requires a grid of uniformly sampled data points.
**FBDD**	Often makes stronger binding ligands from weakly binding fragments. Less time and resource intensive.	Can be used only for small fragments of compound of interest.
**SAR**	Direct observation of target binding to ligand. Several types of NMR experiments are possible.	Inability to distinguish binding modes, difficult to gage the “true” binding site of ligand to protein.
**STD**	Only requires a small amount of sample. Highly reproducible. Allows direct observations of ligand binding.	Only works for ligands with low binding affinity (fast chemical exchange). Inability to distinguish binding modes.
***In cell* NMR**	In vivo studies are possible, can focus on specific cell parts.	Special labeling techniques may be required. Spectra may be more challenging to interpret.
***In silico* + NMR**	Can model protein drug interactions, helps speed up and reduce cost of drug delivery	Protein models need to be validated through experimental approach.
**PNMR**	Can observe proteins interacting with metal ions, long observation distance (10-25 angstroms) between paramagnetic left and nearby atoms.	Paramagnetic left required in the system.
**ALARM NMR**	Elimination of false positives from HTS methods	It requires synthesis of human La antigen protein.
**ssNMR**	Enables the characterization of a chemical compound in a solid-state form such as a tablet/pill. Provides insight into the physical properties of a compound.	Significant broadening of the spectral lineshapes due to anisotropic spin interactions.
**Relaxation editing**	Noticeable difference in spectra of binding and non-binding ligands.	Sets the lower limit of time for which experiments can be performed.
